# Broken adaptive ridge method for variable selection in generalized partly linear models with application to the coronary artery disease data

**DOI:** 10.1016/j.jcmds.2025.100127

**Published:** 2025-10-09

**Authors:** Christian Chan, Xiaotian Dai, Thierry Chekouo, Quan Long, Xuewen Lu

**Affiliations:** a Department of Mathematics and Statistics, University of Calgary, Calgary, AB, Canada; b Department of Mathematics, Illinois State University, Normal, IL 61790, USA; c Division of Biostatistics, School of Public Health, University of Minnesota, Minneapolis, MN 55455, USA; d Department of Biochemistry and Molecular Biology, Department of Medical Genetics, Department of Mathematics and Statistics, Alberta Children’s Hospital Research Institute, Hotchkiss Brain Institute, University of Calgary, Canada

**Keywords:** Bernstein polynomials, BAR regression method, Generalized partly linear models, High-dimensional data, Logistic partly linear model, Coronary artery disease

## Abstract

Motivated by the CATHGEN data, we develop a new statistical method for simultaneous variable selection and parameter estimation in the context of generalized partly linear models for data with high-dimensional covariates. The method is referred to as the broken adaptive ridge (BAR) estimator, which is an approximation of the $L_{0}$-penalized regression by iteratively performing reweighted squared $L_{2}$-penalized regression. The generalized partly linear model extends the generalized linear model by incorporating a non-parametric component, allowing for the construction of a flexible model to capture various types of covariate effects. We employ the Bernstein polynomials as the sieve space to approximate the non-parametric functions so that our method can be implemented easily using the existing R packages. Extensive simulation studies suggest that the proposed method performs better than other commonly used penalty-based variable selection methods. We apply the method to the CATHGEN data with a binary response from a coronary artery disease study, which motivated our research, and obtained new findings in both high-dimensional genetic and low-dimensional non-genetic covariates.

## 1. Introduction

In the era of high technology and supercomputing power, the combination of lower financial costs and greater accessibility to DNA sequencing technology has contributed to the rapid rise in omics research [1]. The high-throughput DNA sequencing equipment produces high-dimensional data, which motivates researchers to identify the genetic variations in the genome that are relevant to the phenotype. In our research, we develop a new statistical method to further decode acquired data and help scientists to find relevant genetic covariates. Variable selection is one of the statistical methods and is an important task when building a statistical model. Given a large number of explanatory variables in a particular study, we want to select the variables that are relevant to the response variable. One way to do this is by using the best subset selection, which is based on the $L_{0}$-regularization. The best subset selection method directly penalizes the cardinality of a model subject to an information criterion, like the AIC [2] and BIC [3]. There are several disadvantages to the $L_{0}$-regularization method, the most important of which is the computational complexity at scales of $2^{p}$, where p is the dimension of the covariates, thus making it computationally expensive for even a moderately large number of covariates. Additionally, Breiman [4] also showed that the $L_{0}$-regularization is unstable in terms of variable selection. Penalty-based variable selection methods were introduced to solve the computational inefficiency of $L_{0}$-regularization. The significance of penalty-based variable selection is the reformulation of the sparse estimation problem into a continuous and nonconvex or convex optimization problem with a smaller number of candidate models. Such methods include the Least Absolute Shrinkage and Selection Operator (LASSO) [5], Smoothly Clipped Absolute Deviation (SCAD) [6], the Elastic-Net penalty [7], the Adaptive LASSO [8], and the Minimax Concave Penalty (MCP) [9].

Recently, the Broken Adaptive Ridge (BAR) regression method has been introduced as an approximation to the $L_{0}$-regularization for variable selection. The BAR regression can be summarized as an iteratively reweighted squared $L_{2}$-penalized regression, where the estimators of the BAR method are taken at the limit of the algorithm. Liu and Liu [10] first considered the implementation of the BAR method under generalized linear models (GLM). Since then, many papers have investigated the BAR method for different models and data types, including the Cox PH model with large-scale right-censored survival data [11], the linear model with uncensored data [12], the additive hazards model with recurrent event data [13], the Cox PH model with interval-censored data [14], the partly linear Cox PH model with right-censored data [15], and the accelerated failure time model with right-censored data [16], among others. Most recently, Mahmoudi and Lu [17] incorporated the BAR method for semi-competing risks data under the illness-death model. Previous works [11–13] have also proved that the BAR method possesses two desired large-sample properties: consistency for variable selection and asymptotic normality, which are called the oracle properties in the literature.

Motivated by the CATHGEN data detailed below, our goal in this research is to extend the BAR method to select important variables in generalized partly linear models (GPLMs) with a large number of genetic covariates, in the presence of some low-dimensional non-genetic covariates. Particularly, we apply the proposed method to select important single nucleotide polymorphisms (SNPs) in a logistic partly linear model in the presence of both categorical and continuous low-dimensional non-genetic covariates, which belongs to a family of generalized partly linear models. In the CATHeterization GENetics (CATHGEN) study, the primary objective was to assess the association of multiple genetic markers with cardiovascular disease phenotypes. The study, conducted by Duke University Medical Centre, collected peripheral blood samples from consenting patients between 2001 and 2012. The follow-up period of the recruited patients was between 2004 and 2014. Aside from the high-dimensional genetic data, low-dimensional baseline clinical and demographical variables were also measured when patients were first recruited to the study. The data can be downloaded from the U.S. National Institute of Health dbGaP data accession number phs000704.v1.p1. We will use the proposed method to analyze the data to identify important SNPs and the associated genes relevant to coronary artery disease (CAD).

The contributions of our work can be summarized into three main aspects. **First**, we develop a new statistical method for simultaneous variable selection and estimation under the context of generalized partly linear models using the BAR method. GPLMs extend GLMs by adding a non-parametric component to them to allow for flexible modeling of linear and non-linear covariate effects. Our method extends the work by Li et al. [18], which incorporates the BAR method under GLM with sparse high-dimensional and massive sample size data for variable selection. Since the low-dimensional covariates in our work contain both categorical and continuous variables, our method treats them separately; only the continuous variables are considered to possess potential non-linear effects. **Second**, we focus specifically on the logistic partly linear regression model, as motivated by the presence of the binary response variable (CAD vs. no CAD) and various types of covariates in the CATHGEN data. We apply our proposed method to the CATHGEN data to identify relevant genetic markers (i.e., SNPs) in high dimensions that contribute to developing CAD. We are also interested in the estimation of the low-dimensional relevant non-genetic covariate effects, which can handle both linear and non-linear covariate effects. **Third**, our method can be easily implemented using existing R packages developed for GLM, since we can use Bernstein polynomials to construct a linear sieve space for estimating the non-parametric functions of the low-dimensional covariates so that the resultant model form mimics a GLM and the existing GLM R packages can be used for estimation and variable selection. We make our code available at https://github.com/chrischan94/GPLM-BAR.

The rest of this article is organized as follows. In the next section, we give a comprehensive introduction to GPLMs, and a detailed explanation of our proposed method and its algorithm. In the section titled *Simulation Studies*, we present the results of our extensive simulation studies, where we compare our method to a few common variable selection methods. In the section titled *Real Data Analysis*, we present the results of the real data analysis of the CATHGEN study and the biological interpretation of the results. In the final section, we conclude our findings in this article and discuss possible future directions for research. The description of the involved algorithm and more simulation results are relegated to the supplementary material.

## 2. Models and methods

### 2.1. Generalized partly linear models

Working under the framework of GLM, consider a random sample $v_{i}={\left(x_{i}^{⊤},y_{i}\right)}^{⊤},i=1,\ldots ,n$, where $y={\left\{y_{1},\ldots ,y_{n}\right\}}^{⊤}$ makes a n×1 response vector and matrix $X={\left\{x_{1},\ldots ,x_{n}\right\}}^{⊤}$ makes a n×p design. The observations $v_{i}={\left(x_{i}^{⊤},y_{i}\right)}^{⊤},i=1,\ldots ,n$ are mutually independent. The distribution of $y_{i}$ conditional on $x_{i}$ is from the exponential family with the following density,

(1) $$f_{y}\left(y_{i};\theta_{i},\phi \right)=\text{exp}\left\{\frac{y_{i}\theta_{i}-b\left(\theta_{i}\right)}{a\left(\phi \right)}+c\left(y_{i},\phi \right)\right\},$$

where a(⋅),b(⋅) and c(⋅,⋅) are known specific functions, b(⋅) is assumed to be twice differentiable, $\theta_{i}$ is the canonical parameter and ϕ denotes the dispersion parameter. Model (1) indicates that $E\left(y_{i}|x_{i}\right)=\mu_{i}=b^{\prime }\left(\theta_{i}\right)$ and $V\left(y_{i}|x_{i}\right)=b^{\prime \prime }\left(\theta_{i}\right)a(\phi )$. Through a link function $g\left(\mu_{i}\right)=\beta_{0}+\beta^{⊤}x_{i}$, the canonical parameter $\theta_{i}$ is connected to $x_{i}$ by a linear combination of the coefficient parameter vector $\beta ={\left\{\beta_{1},\ldots ,\beta_{p}\right\}}^{⊤}$, where $\beta_{0}$ is the intercept parameter. When $g\left(\mu_{i}\right)=\theta_{i}$, it is called the canonical link function. Commonly used canonical link functions in GLMs include the identity function for linear regression, the logit link function for logistic regression and the log function for Poisson regression. Given our observed data, the likelihood function of β for GLMs is

$${ℒ}_{n}\left(\beta ;v_{i}\right)=\prod_{i=1}^{n}f_{y}\left(y_{i};\theta_{i},\phi \right)=\prod_{i=1}^{n}\text{exp}\left\{\frac{y_{i}\theta_{i}-b\left(\theta_{i}\right)}{a(\phi )}+c\left(y_{i},\phi \right)\right\},$$

and the log-likelihood is

$$\ell_{n}\left(\beta \right)=\text{log}\;{ℒ}_{n}\left(\beta ;v_{i}\right)=\sum_{i=1}^{n}\text{log}\;f_{y}\left(y_{i};\theta_{i},\phi \right).$$

GLM assumes a linear relationship between the independent variables ${\left\{x_{1},\ldots ,x_{n}\right\}}^{⊤}$ and the canonical link function. If this assumption is violated for a subset of variables that may have a non-linear relationship with the response, an alternative model form is desirable. Motivated by the CATHGEN data that contains both genetic and non-genetic variables, the covariates can be broadly grouped into three distinct sets: a n×p design matrix $X={\left\{x_{1},\ldots ,x_{n}\right\}}^{⊤}$, a $n\times q_{w}$ design matrix $W={\left\{w_{1},\ldots ,w_{n}\right\}}^{⊤}$, and a $n\times q_{z}$ design matrix $Z={\left\{z_{1},\ldots ,z_{n}\right\}}^{⊤}$. The design matrix X contains the high-dimensional genetic covariates, W contains the low-dimensional and non-genetic categorical covariates, and Z contains the low-dimensional and non-genetic continuous covariates. Then we define the generalized partly linear model as follows, which extends GLM by adding a non-parametric component in the linear predictor,

$$g\left\{E\left(y_{i}|x_{i},w_{i},z_{i}\right)\right\}=\beta^{⊤}x_{i}+\alpha^{⊤}w_{i}+\Psi \left(z_{i}\right),$$

where $\beta ={\left\{\beta_{1},\ldots ,\beta_{p}\right\}}^{⊤},\alpha ={\left\{\alpha_{1},\ldots ,\alpha_{q_{w}}\right\}}^{⊤},\Psi \left(z_{i}\right)=\sum_{j=1}^{q_{z}}\psi_{j}\left(z_{ij}\right),\psi_{j}(\cdot )$’s are unknown smooth functions, which model possible non-linear effects as shown in the analysis of the CATHGEN data. In this model, an intercept parameter $\beta_{0}$ is absorbed into one of the $\psi_{j}$ functions. For identifiability consideration, without loss of generality, we assume $\beta_{0}=0$ and $\psi_{j}\left(M\right.{id}_{j})=0$, where ${Mid}_{j}$ is the middle point of the interval where $\psi_{j}\left(z_{j}\right)$ takes value. For variable selection, the effect of each variable is measured on the same scale, we assume each variable $x_{j},j=1,\ldots ,p$ is normalized such that $\sum_{i=1}^{n}x_{ij}/n=0$ and $\sum_{i=1}^{n}x_{ij}^{2}/n=1$. A special case of GPLM is the logistic partly linear model. Let $\pi \left(y_{i}|x_{i},w_{i},z_{i}\right)=P\left(y_{i}=1|x_{i},w_{i},z_{i}\right)$, then the logistic partly linear model has the model equation given by

$$\text{log}\frac{\pi \left(y_{i}|x_{i},w_{i},z_{i}\right)}{1-\pi \left(y_{i}|x_{i},w_{i},z_{i}\right)}=\beta^{⊤}x_{i}+\alpha^{⊤}w_{i}+\Psi \left(z_{i}\right).$$

For the observations $\left\{u_{i},i=1,\ldots ,n\right\}=\left\{\left\{y_{i},x_{i},w_{i},z_{i}\right\},i=1,\ldots ,n\right\}$, the likelihood function of the logistic partly linear model can be constructed as

$${ℒ}_{n}\left(\alpha ,\beta ,\psi \right)=\prod_{i=1}^{n}\pi {\left(y_{i}|x_{i},w_{i},z_{i}\right)}^{y_{i}}{\left\{1-\pi \left(y_{i}|x_{i},w_{i},z_{i}\right)\right\}}^{1-y_{i}},$$

where $\psi ={\left\{\psi_{1}(\cdot ),\ldots ,\psi_{q_{z}}(\cdot )\right\}}^{⊤}$. From the likelihood function, the log-likelihood can be easily derived as follows

(2) $$\ell_{n}\left(\alpha ,\beta ,\psi \right)=\overset{n}{\underset{i=1}{\sum }}\left[y_{i}\left(\beta^{⊤}x_{i}+\alpha^{⊤}w_{i}+\Psi \left(z_{i}\right)\right)-\text{log}\left(1+\text{exp}\left\{\beta^{⊤}x_{i}+\alpha^{⊤}w_{i}+\Psi \left(z_{i}\right)\right\}\right)\right].$$

Direct estimation of ϑ=(α,β,ψ) in (2) will not be possible because of the presence of the unknown functions $\Psi \left(z_{i}\right)$, which are infinitely dimensional. Hence, approximating the non-parametric part is needed. As the unknown functions $\Psi \left(z_{i}\right)$ are infinitely dimensional, we propose to construct a sieve space to linearize them. To apply the sieve method, we employ the Bernstein polynomials to approximate $\Psi \left(z_{i}\right)$, then the Bernstein polynomials approximation reduces the infinitely dimensional space to a finitely dimensional space. Let Θ denote the parameter space of ϑ where

$$\Theta =\left\{\vartheta =\left(\alpha ,\beta ,\psi_{1},\ldots ,\psi_{q_{z}}\right)\in 𝒜⊗{ℳ}_{1}⊗\cdots ⊗{ℳ}_{q_{z}}\right\},$$

where

$$𝒜=\left\{(\alpha ,\beta )\in R^{q_{w}}\times R^{p},\|\alpha \|+\|\beta \|\leq M\right\},$$

M is a positive constant, and ${ℳ}_{j}$ denotes the collection of all bounded and continuous functions over the range of the observed $z_{j}$ for $j=1,\ldots ,q_{z}$. Subsequently, the sieve space is defined as

$$\Theta_{n}=\left\{\vartheta_{n}=\left(\alpha ,\beta ,\psi_{1n},\ldots ,\psi_{q_{z}n}\right)\in 𝒜⊗{ℳ}_{1n}⊗\cdots ⊗{ℳ}_{q_{z}n}\right\},$$

where

(3) $${ℳ}_{jn}=\left\{\psi_{jn}\left(z_{ij}\right)=\sum_{k=0}^{m_{j}}\gamma_{jk}B_{jk}\left(z_{ij},m_{j},c_{j},u_{j}\right):\sum_{0\leq k\leq m_{j}}\left|\gamma_{jk}\right|\leq M_{jn}\right\},$$

for $j=1,\ldots ,q_{z}$ and $z_{ij}\in \left[c_{j},u_{j}\right],c_{j}<u_{j}$. The Bernstein basis polynomial of $m_{j}$ degree, denoted by $B_{jk}\left(z_{ij},m_{j},c_{j},u_{j}\right)$ in (3), has the equation

Bjkzij,mj,cj,uj=mjkzij−cjuj−cjk1−zij−cjuj−cjmj−k,k=0,…,mj.

Therefore, the sieve log-likelihood of the logistic partly linear model using the Bernstein polynomial to approximate $\Psi \left(z_{i}\right)$ is

(4) $$\ell_{n}\left(\alpha ,\beta ,\gamma \right)=\overset{n}{\underset{i=1}{\sum }}\{y_{i}\left(\beta^{⊤}x_{i}+\alpha^{⊤}w_{i}+\overset{q_{z}}{\underset{j=1}{\sum }}\overset{m_{j}}{\underset{k=0}{\sum }}\gamma_{jk}B_{jk}\left(z_{ij},m_{j},c_{j},u_{j}\right)\right)-\text{log}\left(1+\text{exp}\left\{\beta^{⊤}x_{i}+\alpha^{⊤}w_{i}+\overset{q_{z}}{\underset{j=1}{\sum }}\overset{m_{j}}{\underset{k=0}{\sum }}\gamma_{jk}B_{jk}\left(z_{ij},m_{j},c_{j},u_{j}\right)\right\}\right)\},$$

where $\gamma ={\left\{\gamma_{10},\ldots ,\gamma_{1m_{1}},\ldots ,\gamma_{q_{z}0},\ldots ,\gamma_{q_{z}m_{q_{z}}}\right\}}^{⊤}$.

### 2.2. Simultaneous estimation and variable selection using the GPLM-BAR method

To conduct simultaneous estimation and variable selection in GPLMs, we propose the GPLM-BAR method, which is an iterative method. Following the BAR method by Li et al. [18] for GLM, starting from an initial value vector computed from the following the ridge regression,

(5) $$\left({\overset{ˆ}{\alpha }}^{\left(0\right)},{\overset{ˆ}{\beta }}^{\left(0\right)},{\overset{ˆ}{\gamma }}^{\left(0\right)}\right)=\underset{\alpha ,\beta ,\gamma }{\text{arg}\;\text{min}}\left\{-2\ell_{n}(\alpha ,\beta ,\gamma )+\xi_{n}\sum_{j=1}^{p}\beta_{j}^{2}\right\}.$$

For s≥1, the estimator is iteratively updated by a reweighted squared $L_{2}$-penalized regression

(6) $$\left({\overset{ˆ}{\alpha }}^{\left(s\right)},{\overset{ˆ}{\beta }}^{\left(s\right)},{\overset{ˆ}{\gamma }}^{\left(s\right)}\right)=\underset{\alpha ,\beta ,\gamma }{\text{arg}\;\text{min}}\left\{-2\ell_{n}(\alpha ,\beta ,\gamma )+\lambda_{n}\sum_{j=1}^{p}\frac{\beta_{j}^{2}}{{\left({\overset{ˆ}{\beta }}_{j}^{\left(s-1\right)}\right)}^{2}}\right\},$$

where $\xi_{n}$ and $\lambda_{n}$ are non-negative tuning parameters. The updated step in (6) is continued until a pre-specified convergence criterion is reached, where the estimators are taken at the limit as

$$\hat{\beta }=\underset{s\to \infty }{\text{lim}}{\hat{\beta }}^{\left(s\right)},\;\hat{\alpha }=\underset{s\to \infty }{\text{lim}}{\hat{\alpha }}^{\left(s\right)},\;\hat{\gamma }=\underset{s\to \infty }{\text{lim}}{\hat{\gamma }}^{\left(s\right)}.$$

The implementation of the proposed method indicates the variable selection is done only on high-dimensional covariates $x_{i},i=1,\ldots ,n$, since the penalty is imposed on β only. In addition, the proposed variable selection method can be applied in a similar fashion to the Poisson partly linear regression and the partly linear model for counts and continuous responses, respectively, since they are in the family of GPLMs.

#### 2.2.1. A note on choosing the tuning parameters

**Fig. 1.**
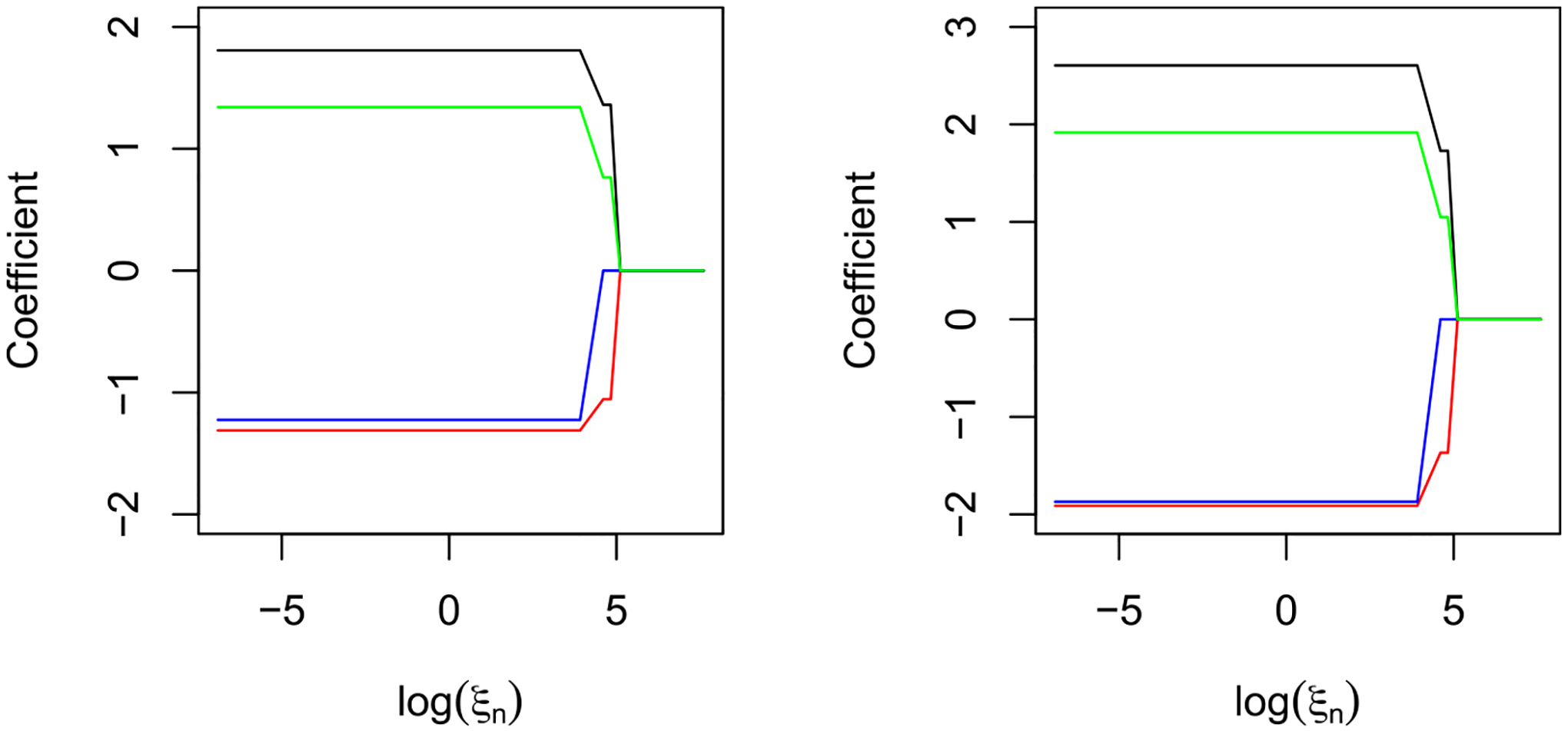
Path plots for the logistic partly linear regression with varying $\xi_{n}$ for a random sample of size n=200 and p=10, using both the BIC (left panel) and AIC (right panel) penalties.

Choosing the optimal values of tuning parameters is crucial for penalty-based variable selection methods, as it greatly affects the variable selection accuracy. In the absence of an external validation set, common methods to find the optimal values of the tuning parameters include the k-fold cross-validation (CV) method. The optimal tuning parameter value is the one that minimizes a criterion. Typically, this is the mean squared error for continuous outcome, or deviance for a binary outcome. However, doing this only adds to the computational complexity, and it is not ideal for larger datasets. In the GPLM-BAR algorithm, we have two tuning parameters: $\xi_{n}$ and $\lambda_{n}$. Unless the value of $\xi_{n}$ chosen is large, it is empirically shown that the value chosen is inconsequential on the estimation of β, as seen in Fig. 1. Hence, $\xi_{n}$ is set to a relatively small value. For $\lambda_{n}$ in the Cox-BAR regression, it has been argued by Kawaguchi et al. [11] that it can be fixed. One example is fixing $\lambda_{n}=\text{log}(n)$, which corresponds to the BIC penalty. Another example is to fix $\lambda_{n}=2$, which corresponds to the AIC penalty. In our method, both the AIC and BIC penalties are considered.

#### 2.2.2. Computational aspects for GPLM-BAR

Except under the linear model, numerical approximation methods such as the Newton–Raphson algorithm are integrated into the implementation of the BAR penalty for simultaneous variable selection and estimation. When both the number of covariates and sample size are small, calculating the partial gradient vector and Hessian matrix at each iteration of the BAR algorithm is computationally feasible. However, when both the number of covariates and sample size become moderately large, numerical approximation becomes non-scalable because of the high computational costs and the numerical instability. Alternative optimization techniques for parameter estimation under large-scale regularization and regression problems [19,20] have been developed. The algorithm by Zhang and Oles [19] called column relaxation of logistic loss (CLG) can be classified as a cyclic coordinate descent algorithm.

The R package BrokenAdaptiveRidge [11] was created to implement BAR regression for GLM and the Cox model, which are linear models. Since we have reparameterized our GPLM into a form of GLM in (4), we are able to directly use the package to conduct variable selection and estimation under the context of GPLM. This package uses the R package Cyclops [21] for efficient implementation of the iterative method as described in Kawaguchi et al. [11]. The computation in the package is done by the cyclic coordinate descent algorithm. We describe this algorithm for the GPLM-BAR regression in the supplementary material.

## 3. Simulation studies

In this section, we present the results of a comprehensive simulation study in five scenarios to demonstrate the effectiveness of our proposed method. The first and second scenarios assess the performance under strong signals and weak signals, respectively, under the setting of the logistic partly linear model. The third scenario performs a simulation study for the ultra-high-dimensional scenario, where p>n. The fourth scenario uses the selected model in the real data analysis section for the CATHGEN data as a basis to simulate data, then assesses the performance of our method. The fifth scenario shows the performance under the setting of the Poisson partly linear model. The results for the fourth and fifth scenarios are relegated to the supplementary material.

### Scenario 1: Strong signals in the logistic partly linear model

In this scenario, let $q_{z}=5$ and $q_{w}=4$, and the number of non-zero elements q=5 in the true parameter p-vector $\beta_{0}$, for various values of p. We generate the n×p design matrix X from a multivariate normal distribution with mean zero and variance–covariance matrix $\Sigma_{X}$, where the (i,j)th entry of it is $\rho^{\left|i-j\right|}$. We fix ρ=0.25. We first consider large effects, i.e., large values of β, where the true value of β is $\beta_{0}=\{1,-1,0,\ldots ,0,-1,0.75,0.75{\}}^{⊤}$. We also generate a $n\times q_{w}$ design matrix W from independent Bernoulli distributions, with the same probability of success π=0.5. And, the true value of α is $\alpha_{0}=\{1,-0.5,-0.5,0.75,-1{\}}^{⊤}$. Independently from X and W, we also generate a $n\times q_{z}$ design matrix Z, where we draw $z_{1}$ from the uniform distribution over (1,5), $z_{2}$ and $z_{3}$ independently from the standard uniform distribution, and $z_{4}$ from the uniform distribution over (−3, 1). By setting the non-linear functions to be $\psi_{1}\left(z_{i1}\right)=0.1{\left(z_{i1}-3\right)}^{2},\psi_{2}\left(z_{i2}\right)=0.2\left(\text{cos}\left(2\pi z_{i2}\right)+1\right),\psi_{3}\left(z_{i3}\right)=0.2\;\text{sin}\left(2\pi z_{i3}\right)$, and $\psi_{4}\left(z_{i4}\right)=0.2{\left(z_{i4}+1\right)}^{3}$, respectively, we generate $y_{i}$ from the Bernoulli distribution with probability $\pi_{i}$, where $\pi_{i}=1/\left(1+\text{exp}\left\{-\beta_{0}^{⊤}x_{i}-\alpha_{0}^{⊤}w_{i}-\sum_{j=1}^{4}\psi_{j}\left(z_{ij}\right)\right\}\right)$. The chosen non-linear functions have two common properties: (1) they are symmetric at the midpoint of the interval of their domains, (2) the values of the functions are zero at the midpoint for identifiability. We consider two different sample sizes n=600 and 800, and two different numbers of high-dimensional covariates p=300 and 450. Each combination is replicated 500 times. The number of basis functions for all non-linear functions is set at $m_{j}+1=3+1=4$, since more than four basis functions only add to the computational complexity while only marginally improving the approximation of $\psi_{j}(\cdot )$, conversely having fewer than four basis functions will not approximate $\psi_{j}(\cdot )$ well.

In the simulation studies, we compare our method with those of LASSO and Adaptive LASSO as well as the SCAD [6] and MCP methods [9]. We use the R package splines2 [22] to generate the Bernstein polynomials. The LASSO and Adaptive LASSO methods are implemented using the R package glmnet [23,24], and the SCAD and MCP methods using the R package ncvreg [25]. The hyperparameters of the SCAD and MCP methods were both set to 2. To evaluate the estimation accuracy, we compute the median mean squared error (MMSE), where the mean squared error is defined as ${\left(\overset{ˆ}{\beta }-\beta_{0}\right)}^{⊤}\Sigma_{X}\left(\overset{ˆ}{\beta }-\beta_{0}\right)$. For the GPLM-BAR method, we fix $\lambda_{n}$ to two values, $\lambda_{n}=2$ and $\lambda_{n}=\text{log}(n)$, which correspond to the AIC and BIC penalties, respectively. Since the value of $\xi_{n}$ was shown to have an inconsequential effect on estimation, we set $\xi_{n}=1$. For the other methods, we use 10-fold CV method to select the optimal value. To evaluate the selection accuracy, we compute the average number of true positives (TP), the average number of false positives (FP), the total misclassification rate (MC) which is the sum of the average number of FP and the average number of false negative (FN), the frequency of true model (TM) selected, and the average estimated size of the model (MS), where MS = TP + FP.

From Table 1, it can be seen that the GPLM-BAR method outperforms the four competing methods. Although the average number of TP may be lower than that of the four competing methods when the BIC penalty is used, it does produce the smallest misclassification error. The SCAD and MCP methods perform better than the LASSO and ALSSO methods, but they produce larger MMSE values. It is interesting to observe a trade-off between the AIC and BIC penalties, where estimation accuracy is improved with the AIC penalty, contrasting with the better variable selection results obtained with the BIC penalty. This is explained by the larger tuning parameter in the BIC penalty, which shrinks the relatively smaller signals in β to zero, thus causing a larger estimation bias. In addition, the estimation results of α are reported in the Supplementary Material Table 3, where the estimates of our method are the best.

**Fig. 2.**
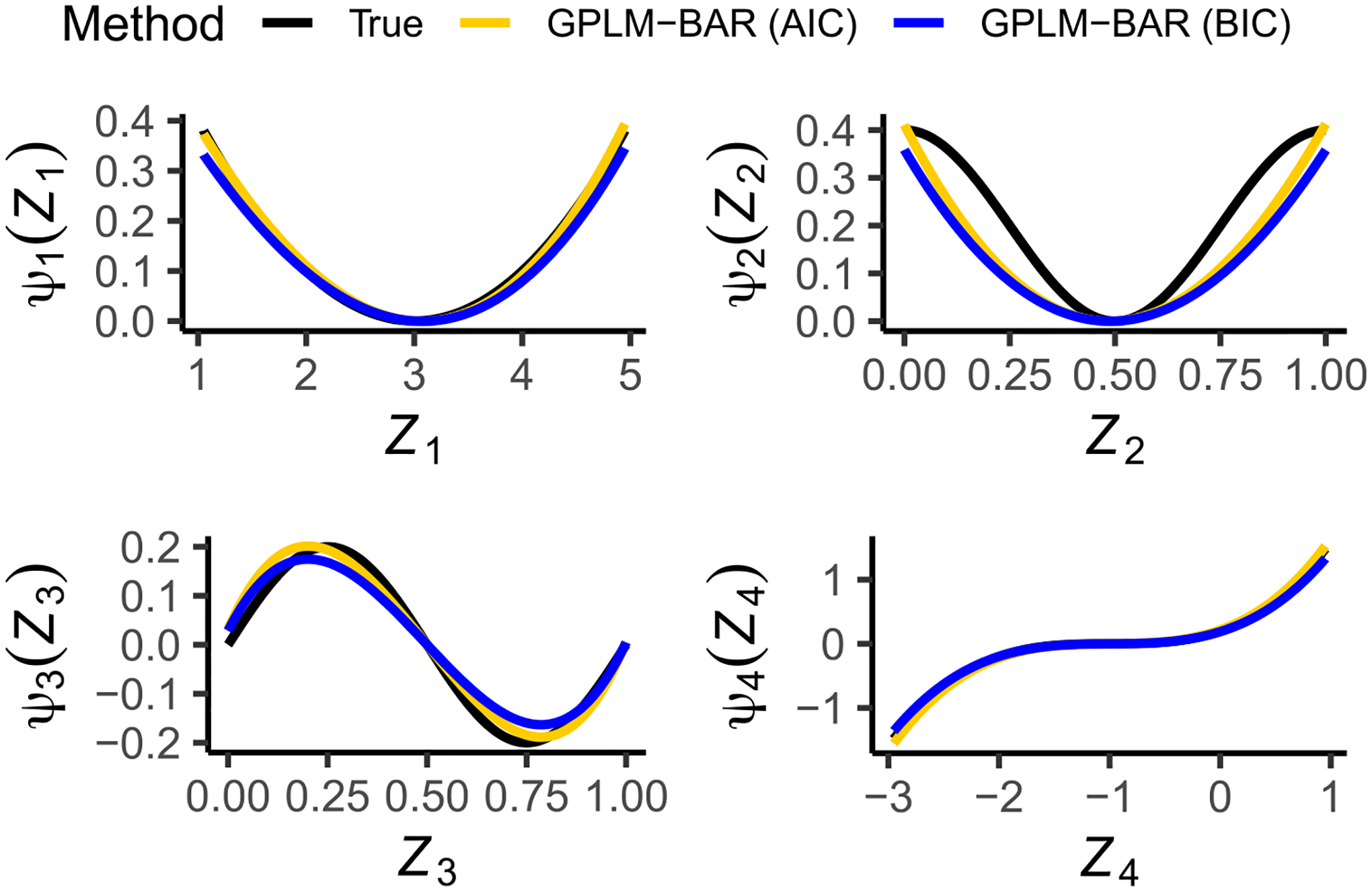
Estimated nonlinear covariate effects $\psi_{j}\left(\cdot \right),j=1,2,3,4$, for Scenario 1,p=300 and n=600. The true functions are: $\psi_{1}\left(z_{1}\right)=0.1{\left(z_{1}-3\right)}^{2}$ (top left), $\psi_{2}\left(z_{2}\right)=0.2\left(\text{cos}\left(2\pi z_{2}\right)+1\right)$ (top right), $\psi_{3}\left(z_{3}\right)=0.2\;\text{sin}\left(2\pi z_{3}\right)$ (bottom left), $\psi_{4}z_{4}=0.2{\left(z_{4}+1\right)}^{3}$ (bottom right).

We are also interested in the estimation of non-linear covariate effects $\psi_{j}(\cdot )$ using the GPLM-BAR method. The estimated curves are shown in Fig. 2, which compares the averaged estimates of each of the four non-linear functions to the true function. Two observations are made. First, the Bernstein polynomial using three basis functions to approximate each $\psi_{j}(\cdot )$ is satisfactory, where the general shape of each function is captured well. Second, different $\lambda_{n}$ tuning methods give slightly different estimates of $\psi_{j}(\cdot )$, as the BAR method with the BIC penalty (blue curve) produces more biases than the AIC penalty (yellow curve). The GPLM-BAR also performs well for the other three combinations of n and p (see Supplementary Material Figures 1–3).

### Scenario 2: Strong and weak signals in the logistic partly linear model

We perform another scenario where some signals of the non-zero entries in β are weaker than those in Scenario 1. Here, we fix q=5, and the true value of β is $\beta_{0}=\{1,-0.5,0,\ldots ,0,-1,0.4,0.75{\}}^{⊤}$. The true values of α and the non-linear functions are the same as in Scenario 1. From Table 2, the GPLM-BAR method with the AIC penalty outperforms the other competing methods. However, in comparison to the results in Scenario 1, the selection and estimation accuracies become worse, because the weaker signals in β have a greater tendency to be shrunk to zero. Using the GPLM-BAR method, the estimation results of the non-linear covariate effects (Supplementary Material Figures 4–7) and α are satisfactory (Supplementary Material Table 4).

It is interesting to compare the computational time of the considered methods. In general, the proposed GPLM-BAR method uses the least time. Using Scenario 1 as an example, we report the computational time for each method in the Supplementary Material Table 5, which shows our method is computationally faster than the competing methods that we considered. It is roughly two times faster than the ALASSO method, eight times faster than the SCAD method, and more than ten times faster than the MCP.

### Scenario 3: High-dimensional scenario

We perform a simulation study for the high-dimensional scenario, where p>n. As in the first two scenarios, we let $q_{z}=5,q_{w}=4$, and the number of non-zero elements q=5 in $\beta_{0}$. We generate the design matrices X,W, and Z in the same way as in Scenarios 1 and 2. The true values for β are $\beta_{0}=\{2,-2,0,\ldots ,0,-2,1.5,1.5{\}}^{⊤}$. To select the tuning parameter $\lambda_{n}$ in this case, Fan and Tang [26] proposed a generalized information criterion (GIC), and Wang et al. [27] proposed a high-dimensional BIC (HBIC), both used $\lambda_{n}=\text{log}\{\text{log}(n)\}\text{log}(p)$. Based on the setting in Scenario 1, we generate data for the combinations of (n,p), where n=600 and n=800,p=2000 and n=4000, respectively. We also include AIC and BIC for comparison with HBIC.

From Table 3, the GPLM-BAR method with the BIC penalty outperforms the same method with the HBIC penalty, both from the perspective of variable selection and estimation. We repeated this scenario when the non-zero signals become weaker and are the same as the $\beta_{0}$ in Scenario 2, where the results summarized in the Supplementary Material Table 6 show a similar pattern. We conclude that the BIC penalty is robust to the strength of the signals in β when the GPLM-BAR method is used for variable selection in high dimensions.

## 4. Real data analysis

Coronary artery disease (CAD) is a major disease that inflicts death, and is one of the biggest causes of death globally [28]. Environmental factors that contribute to CAD are typically age, smoking status, obesity and lifestyle choices. However, genetic factors play a role in death due to CAD, especially in younger patients [29].

We apply our proposed method to the CATHGEN data, which was downloaded from dbGaP, with accession number phs000704.v1.p1. The study collected peripheral blood samples from consenting patients who were undergoing cardiac catheterization at Duke University Medical Center from 2001 to 2011. A total of 1327 patients were recruited and followed up between 2004 and 2014. The binary response variable is the affection status, where the stratification criteria are defined in Shah et al. [30]. The high-dimensional design matrix contains 13 991 columns of SNPs belonging to 331 genes that have been associated with CAD using Ingenuity Pathway Analysis [31]. In addition to the SNPs, there are ten clinical and demographic variables in the data. These variables include age (Mean = 57.0, SD = 11.6), BMI (Mean = 30.8, SD = 7.8), smoking status (671 cases out of 1327), race (897 Caucasian-Americans, 274 African-Americans and 156 Asian-Americans), hypertension status (900 cases out of 1327), diabetes status (379 cases out of 1327), hypercholesterolemia status (745 cases out of 1327), sex (684 males and 643 females), number of diseased vessels and history of myocardial infarction (HXMI) (277 cases out of 1327). All clinical and demographic variables of each subject were measured when they were included in the study. We exclude the number of diseased vessels from further analysis because of conversion issues when fitting the univariate logistic regression model.

**Fig. 3.**
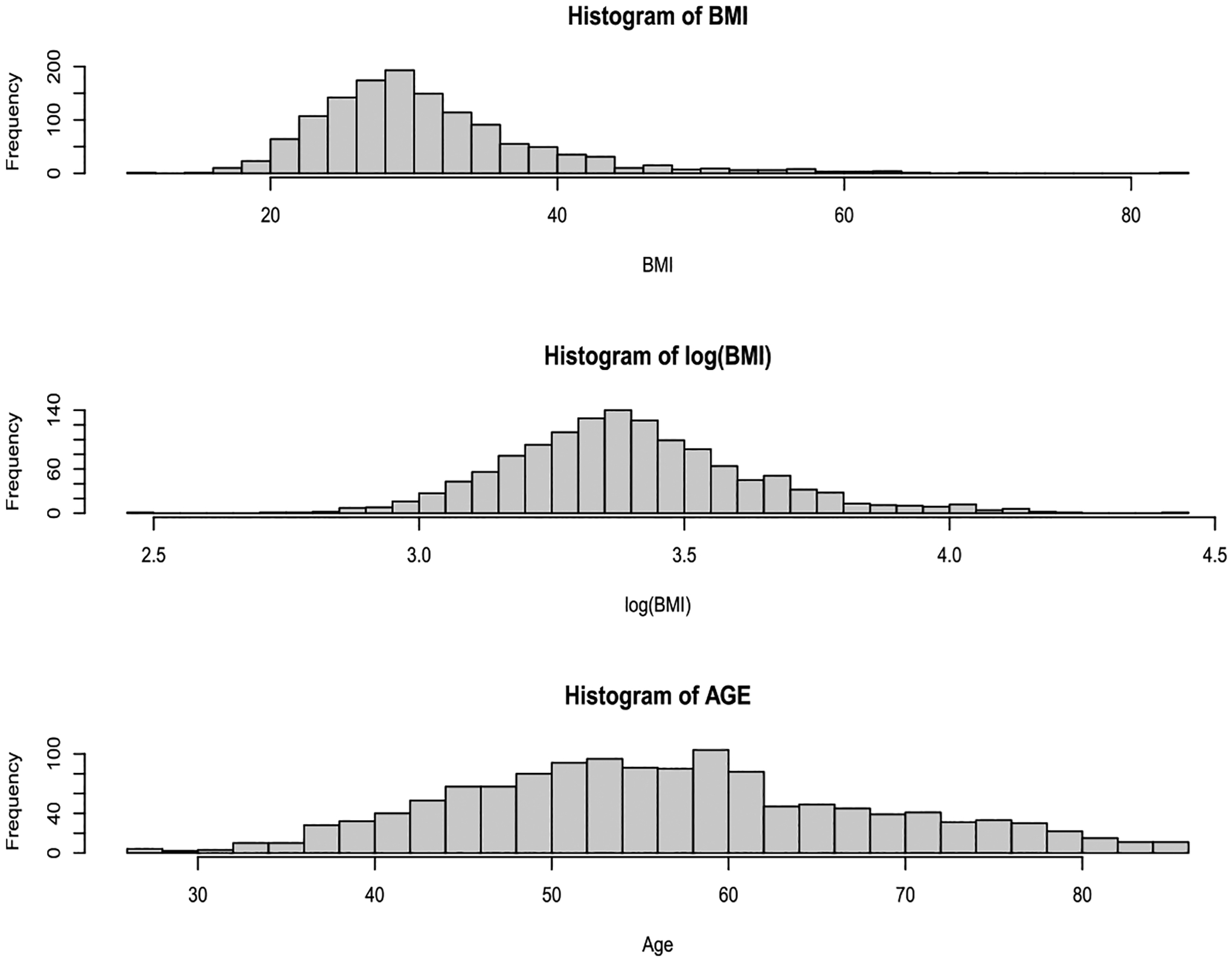
The histograms of the BMI on the original scale (top panel), BMI on the log-transformed scale (middle panel), and the histogram of age on the original scale (bottom panel).

In Fig. 3, the distribution of age is symmetrical on the original scale. However, the distribution of the BMI on the original scale is right-skewed. The natural logarithm transformation of it fixes the skewness. Thus, we decided to use the BMI on the log scale for further analysis. From Table 4, when fitting age and its second-order polynomial term in the logistic regression model, both terms are found to be statistically significant. Likewise, when fitting the log-transformed BMI and its second-order polynomial term in the logistic regression model, both terms are also significant. The results in Table 4 indicate that age and log-transformed BMI have a non-linear effect on the odds ratio of developing CAD. However, the functional form of the effect is unknown, and this motivates us to consider a logistic partly linear regression model.

Noticing that there are 13991 SNPs in the original data set, which is greater than the sample size n=1327. When we apply our GPLM-BAR method with BIC and HBIC to tune $\lambda_{n}$, none of the SNPs are selected, which indicates that the potential effects of the SNPs may be too weak to be detected. We then pre-screen the candidate SNPs to reduce the dimension of the SNPs. First, we remove SNPs with a minor allele frequency (MAF) of less than 0.1. Second, we further reduce the number of SNPs by performing univariate logistic regression, only selecting SNPs with a p-value less than 0.1. At the end, a total number of 1242 SNPs with a p-value less than 0.1 are retained for analysis.

**Fig. 4.**
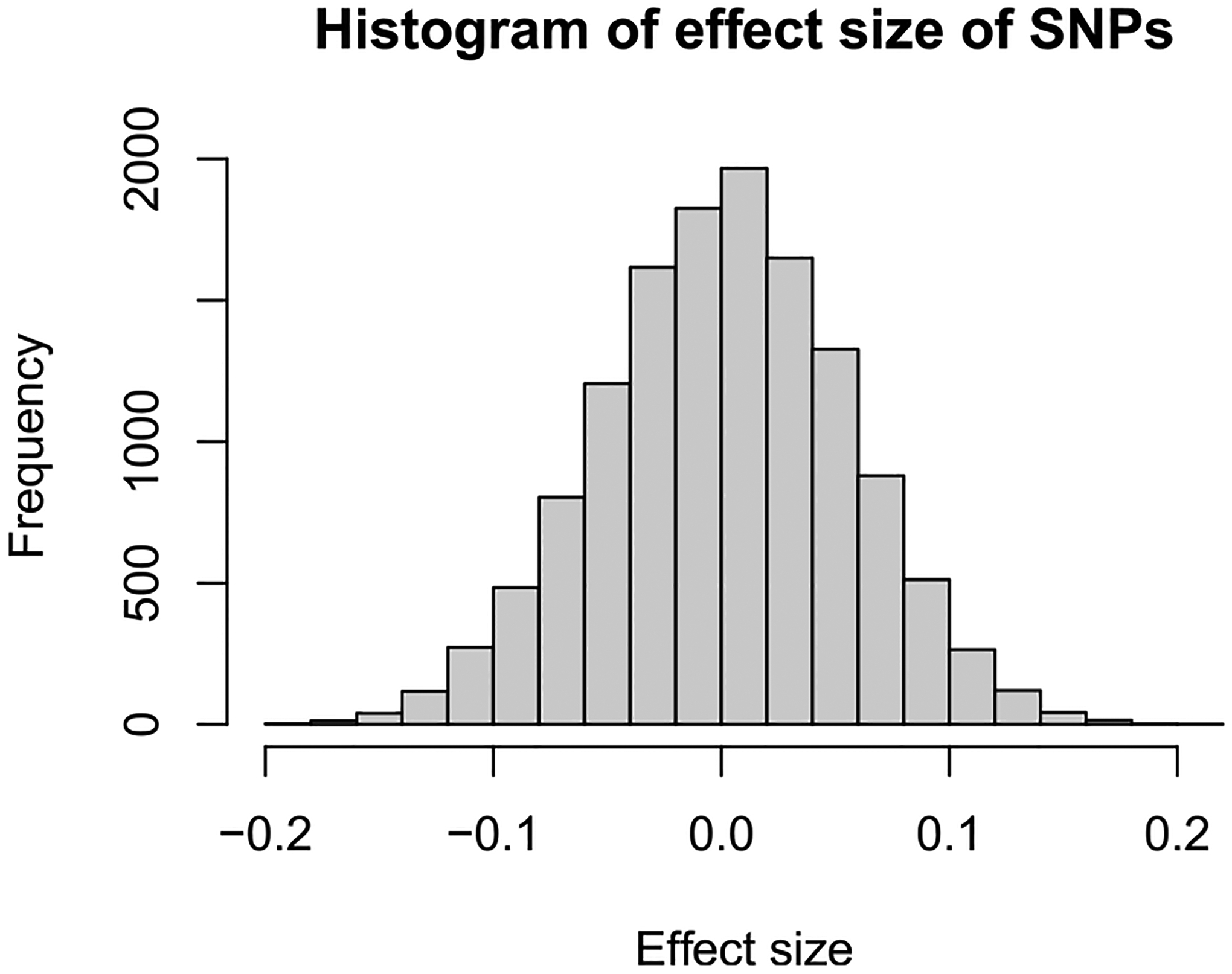
Histogram of the estimated effect sizes of SNPs using univariate logistic regression.

Because the individual estimated effect sizes of the SNPs using univariate logistic regression are small, as shown in Fig. 4, we decide to use AIC to choose the tuning parameters in GPLM-BAR. The value of $\xi_{n}$ and the number of basis functions are kept the same as in the simulation study. The tuning parameter values for the LASSO and Adaptive LASSO methods are chosen by 5-fold cross-validation. In Table 5, the estimated effects of the categorical clinical variables obtained from the GPLM-BAR method have a larger magnitude. The results in Table 5 indicate a positive risk association for hypertension, diabetes, hypercholesterolemia, smoking and HXMI, where HXMI is the strongest clinical indicator on the risk of developing CAD. We use the bootstrap method with 100 random bootstrap samples to obtain the estimated standard error in parentheses in Table 5. The GPLM-BAR method identified the fewest number of SNPs that contribute to CAD. Specifically, the GPLM-BAR identified 19 different SNPs that are associated with 17 unique genes, the LASSO and Adaptive LASSO methods identified 199 SNPs and 228 SNPs, respectively.

From the genes identified using GPLM-BAR, *RBFOX1* is found to be associated with blood pressure and heart failure through transcriptome profiling [32]. *CDH13* has been shown to be associated with blood cholesterol and CAD through a genome-wide association study undertaken in the British population [33]. *F10* is associated with the lowering levels of coagulation factor X, which is protective against ischemic heart disease [34]. *GABRG3* has been shown to be associated with the density of dodecanedioic acid, which plays a role in regulating blood sugar level [35]. *ABCA1* has been shown to be associated with altered lipoprotein levels, which results in an increased risk for CAD [36]. *IL1B* belongs to the wider family of *IL1* genes which is associated with coronary heart disease [37–39]. Certain subtypes of the *APOE* gene are identified associated with lipid levels and coronary risk [40]. We report the complete set of selected SNPs and genes in the supplementary material Table 10.

**Fig. 5.**
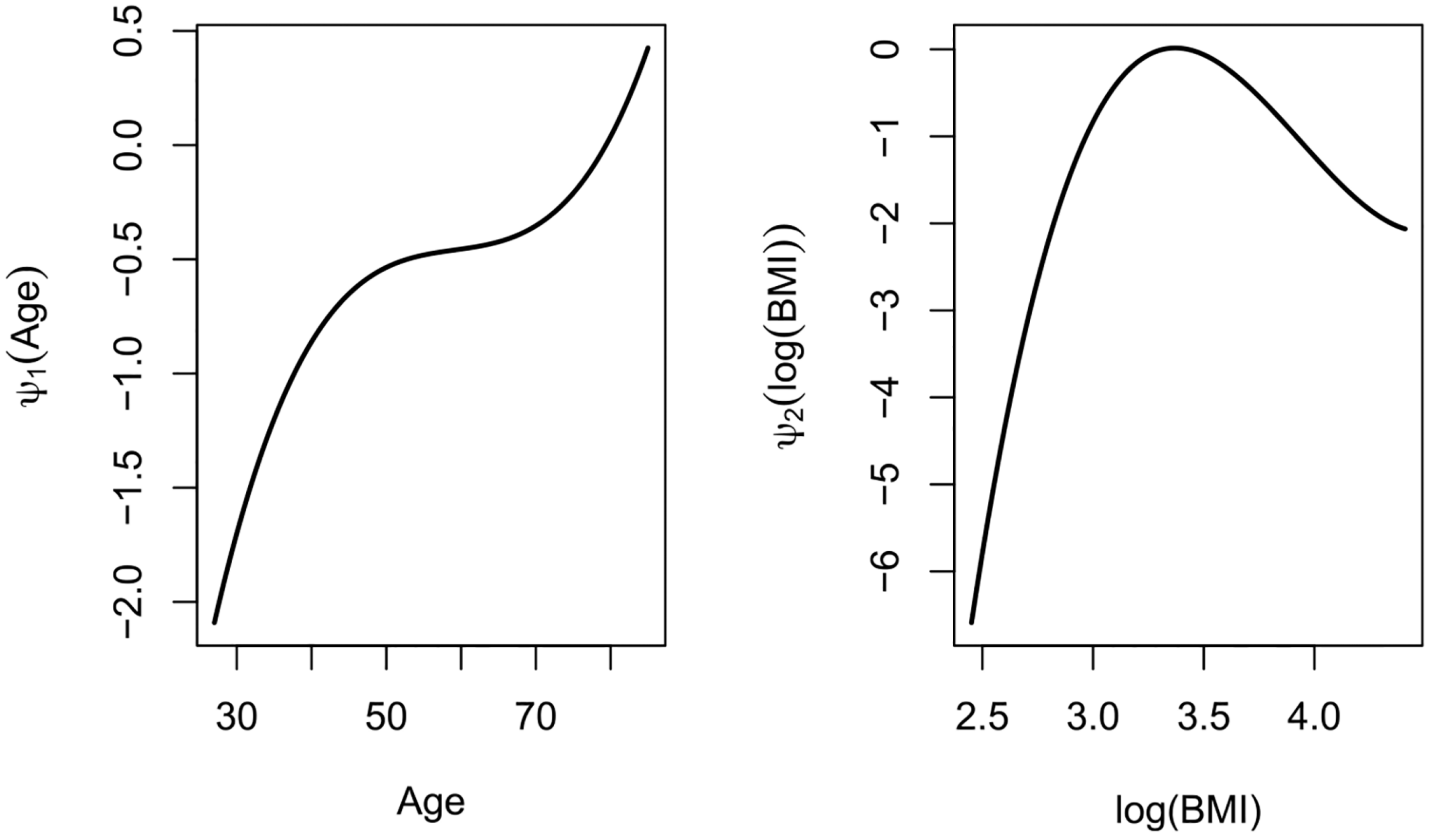
Estimated covariate effects of age (left panel) and log(BMI) (right panel).

In addition to the results in Table 5, one can observe our method using Bernstein polynomials approximation has showed that the effects of age and BMI are non-linear, as seen in Fig. 5. The plot on the left in Fig. 5 shows that the risk of developing CAD increases non-linearly with age, and the plot on the right shows that the risk of developing CAD increases with BMI on the natural logarithm scale until 3.5. After this cutoff point, it then decreases. The unusual trend seen for BMI can be partially explained by the lack of data when BMI > 35 or log(BMI) > 3.5, as the BMI of the majority of patients recruited to this study falls between 15 and 35 on the raw scale. Both plots in Fig. 5 were centered at their respective midpoints of the intervals, and the plot that shows the estimated non-linear effect of age absorbed the intercept term.

## 5. Discussion and conclusion

In this article, we have proposed a new approach for simultaneous variable selection and estimation under the context of GPLM, with a focus on the logistic partly linear regression model. Our proposed approach was motivated by the CATHGEN study, where the data contain both high-dimensional genetic covariates and low-dimensional clinical and demographical covariates. We considered GPLM as it grants us the flexibility to model possible non-linear covariate effects. We employed the Bernstein polynomials to approximate the non-parametric component of the model, where it has several advantages over other approximation methods like splines and piecewise functions. First, unlike the piecewise functions, the Bernstein polynomials are differentiable and continuous everywhere. This is desirable as the first and second derivatives are calculated in each iteration of our algorithm. Second, the Bernstein polynomials possess computational scalability and optimal shape-preserving property for all approximating polynomials [41]. Third, the Bernstein polynomials do not require specification of the number of interior knots and their locations, unlike B-splines. From the results of our comprehensive simulation studies, we observe that our proposed method outperforms common variable selection methods under a few practical scenarios. Our method incorporating the BAR penalty produced the lowest total misclassification rate and the highest frequency of the true model selected, which is consistent with other empirical studies conducted by authors who also employed the BAR penalty [12,14,16]. Our method was also able to accurately estimate the true non-linear functions. As an application, we applied our proposed method to the CATHGEN data, where certain SNPs and genes were found to have a relevant contribution to CAD, which is consistent with other variable selection methods applied to this data [42,43].

There are several directions one can take from our research. Since our focus is variable selection in high-dimensional variables that appear in the linear part, and the dimension of the nonlinear covariates is small, we only considered variable selection in the linear part of the model. As a referee suggested, it would be interesting to see how to incorporate penalization on the gamma coefficients from the Bernstein polynomials, so that one can select significant variables simultaneously in both the linear and additive nonparametric parts. To do that, Huang et al. [44] proposed to use splines to approximate nonlinear functions and applied the adaptive group Lasso to select nonzero components in nonparametric additive models. Li et al. [45] extended their method to additive partially linear models. Using a similar approach, one could also select significant variables simultaneously in both the linear and additive nonparametric parts in our generalized partly linear models. The CATHGEN data also contains right-censored survival information. Under the context of survival models, it would be of interest to investigate which relevant genetic markers affect the survival probability, and the possible homogeneity or heterogeneity of the two sets of genetic markers selected based on the two different response outcomes and their biological interpretations. Additionally, choosing the optimal tuning parameter poses a significant challenge to researchers. The mixture of weak signals with strong signals poses a noteworthy problem to researchers and requires more thorough investigation.

## Supplementary Material

Supplement

## Figures and Tables

**Table 1:** Variable selection and estimation results over 500 replications for Scenario 1. Standard deviations of the MMSE are in parentheses. MMSE: Median Mean Squared Error; TP: True Positive; FP: False Positive; MS: Model Size; MC: Total Misclassification = FN + FP; TM: Frequency of True Model selected. Bold values indicate the method that produces the best result.

Method	MMSE	TP	FP	MS	MC	TM
n=600,p=300						
BAR(AIC)	**0.16(0.11)**	**5**	1.25	6.25	1.25	29%
BAR(BIC)	0.28(0.28)	4.75	**0**	4.75	**0.25**	**76%**
LASSO	1.01(0.25)	**5**	3.83	8.83	3.83	16%
ALASSO	0.45(0.24)	4.99	2.47	7.46	2.48	51%
SCAD	2.97(0.17)	4.77	0.17	**4.94**	0.40	71%
MCP	2.53(0.07)	4.96	0.87	5.83	0.91	57%
Oracle	0.08(0.09)	5	0	5	0	100%
n=800,p=300						
BAR(AIC)	**0.11(0.08)**	**5**	1.29	6.29	1.29	30%
BAR(BIC)	0.16(0.14)	4.96	**0**	4.96	**0.04**	**96%**
LASSO	0.86(0.21)	**5**	3.69	8.69	3.69	22%
ALASSO	0.36(0.22)	**5**	2.30	7.30	2.30	62%
SCAD	2.93(0.18)	4.88	0.11	**4.99**	0.23	86%
MCP	2.53(0.05)	4.99	0.77	5.76	0.78	74%
Oracle	0.05(0.06)	5	0	5	0	100%
n=600,p=450						
BAR(AIC)	**0.22(0.16)**	**5**	2.07	7.07	2.07	14%
BAR(BIC)	0.28(0.27)	4.74	**0**	4.74	**0.26**	**75%**
LASSO	1.04(0.24)	**5**	5.26	10.26	5.26	10%
ALASSO	0.40(0.21)	**5**	6.86	11.86	6.86	27%
SCAD	2.97(0.19)	4.78	0.47	**5.25**	0.69	67%
MCP	2.53(0.06)	4.97	1.29	6.26	1.32	45%
Oracle	0.08(0.09)	5	0	5	0	100%
n=800,p=450						
BAR(AIC)	**0.15(0.09)**	**5**	2.00	7.00	1.99	12%
BAR(BIC)	0.17(0.13)	4.96	**0**	4.96	**0.04**	**96%**
LASSO	0.89(0.20)	**5**	4.55	9.55	4.55	14%
ALASSO	0.33(0.19)	**5**	4.51	9.51	4.51	47%
SCAD	2.89(0.18)	4.87	0.13	**5.00**	0.26	83%
MCP	2.53(0.05)	4.99	0.84	5.83	0.85	71%
Oracle	0.05(0.06)	5	0	5	0	100%

**Table 2:** Variable selection and estimation results over 500 replications for Scenario 2. Standard deviations of the MMSE are in parentheses. MMSE: Median Mean Squared Error; TP: True Positive; FP: False Positive; MS: Model Size; MC: Total Misclassification = FN + FP; TM: Frequency of True Model selected. Bold values indicate the best method.

Method	MMSE	TP	FP	MS	MC	TM
n=600,p=300						
BAR(AIC)	**0.20(0.14)**	**4.66**	1.32	**5.98**	1.66	**19%**
BAR(BIC)	0.49(0.16)	3.18	**0**	3.18	1.82	1%
LASSO	0.81(0.22)	4.39	2.98	7.37	3.59	7%
ALASSO	0.38(0.20)	4.39	4.27	8.66	4.88	10%
SCAD	2.21(0.11)	3.41	0.03	3.44	**1.62**	5%
MCP	1.88(0.07)	4.38	1.73	6.11	2.35	9%
Oracle	0.07(0.07)	5	0	5	0	100%
n=800,p=300						
BAR(AIC)	**0.11(0.09)**	**4.88**	1.21	**6.09**	**1.33**	**31%**
BAR(BIC)	0.41(0.13)	3.47	**0**	3.47	1.53	4%
LASSO	0.67(0.16)	4.72	2.56	7.28	2.84	20%
ALASSO	0.30(0.16)	4.63	2.99	7.62	3.36	26%
SCAD	2.20(0.10)	3.49	0.01	3.50	1.52	6%
MCP	1.88(0.05)	4.66	1.62	6.28	1.96	16%
Oracle	0.05(0.04)	5	0	5	0	100%
n=600,p=450						
BAR(AIC)	**0.23(0.15)**	**4.65**	1.76	**6.41**	2.11	**12%**
BAR(BIC)	0.50(0.18)	3.17	**0**	3.17	1.83	0%
LASSO	0.89(0.23)	4.32	3.10	7.42	3.78	7%
ALASSO	0.41(0.21)	4.39	7.84	12.23	8.45	4%
SCAD	2.21(0.10)	3.45	0.07	3.52	**1.62**	6%
MCP	1.89(0.07)	4.32	1.96	6.28	2.64	7%
Oracle	0.07(0.07)	5	0	5	0	100%
n=800,p=450						
BAR(AIC)	**0.14(0.10)**	**4.89**	1.91	6.80	2.02	13%
BAR(BIC)	0.41(0.14)	3.47	**0**	**3.47**	**1.53**	4%
LASSO	0.72(0.17)	4.69	2.99	7.68	3.30	13%
ALASSO	0.29(0.15)	4.74	7.19	11.93	7.45	**16%**
SCAD	2.20(0.10)	3.44	0.01	3.45	1.57	5%
MCP	1.87(0.04)	4.67	2.09	6.76	2.42	14%
Oracle	0.05(0.05)	5	0	5	0	100%

**Table 3:** Variable selection and estimation results over 500 replications for Scenario 3. Standard deviations of the MMSE are in parentheses. MMSE: Median Mean Squared Error; TP: True Positive; FP: False Positive; MS: Model Size; MC: Total Misclassification = FN + FP; TM: Frequency of True Model selected. Bold values indicate the best method.

Method	MMSE	TP	FP	MS	MC	TM
n=600,p=2000						
BAR (AIC)	**0.29(0.27)**	**5**	0.97	5.97	0.97	37%
BAR (BIC)	0.51(0.34)	**5**	**0**	**5**	**0**	**100%**
BAR (HBIC)	3.44(0.27)	4.69	**0**	4.69	0.31	71%
LASSO	4.71(0.67)	**5**	14.95	19.95	14.95	1%
ALASSO	1.28(0.68)	**5**	16.77	21.77	16.77	12%
SCAD	12.27(0.42)	4.90	**0**	4.90	0.10	90%
MCP	11.54(0.37)	4.52	0.62	5.14	1.10	51%
Oracle	0.20(0.34)	5	0	5	0	100%
n=800,p=2000						
BAR (AIC)	0.35(0.26)	**5**	2.11	7.11	2.11	10%
BAR (BIC)	**0.32(0.24)**	**5**	**0**	**5**	**0**	**100%**
BAR (HBIC)	1.79(0.49)	4.99	**0**	4.99	0.01	99%
LASSO	3.95(0.62)	**5**	15.60	20.60	15.60	1%
ALASSO	0.88(0.60)	**5**	15.42	20.42	15.42	19%
SCAD	12.21(0.39)	4.93	**0**	4.93	0.07	93%
MCP	11.56(0.31)	4.58	0.76	5.34	1.18	52%
Oracle	0.14(0.25)	5	0	5	0	100%
n=600,p=4000						
BAR (AIC)	**0.22(0.35)**	**5**	0.27	5.27	0.27	76%
BAR (BIC)	0.49(0.33)	**5**	**0**	**5**	**0**	**100%**
BAR (HBIC)	4.76(1.46)	4.38	**0**	4.38	0.62	46%
LASSO	4.82(0.78)	**5**	21.94	26.94	21.94	2%
ALASSO	1.23(0.52)	**5**	35.19	40.19	35.19	5%
SCAD	12.29(0.40)	4.90	**0**	4.90	0.10	90%
MCP	11.56(0.39)	4.50	1.27	5.77	1.77	44%
Oracle	0.21(0.56)	5	0	5	0	100%
n=800,p=4000						
BAR (AIC)	**0.19(0.15)**	**5**	0.69	5.69	0.69	47%
BAR (BIC)	0.34(0.23)	**5**	**0**	**5**	**0**	**100%**
BAR (HBIC)	2.26(0.57)	4.97	**0**	4.97	0.03	97%
LASSO	4.33(0.59)	**5**	18.71	23.71	18.71	1%
ALASSO	0.88(0.41)	**5**	32.94	37.94	32.94	7%
SCAD	12.21(0.38)	4.93	**0**	4.93	0.07	94%
MCP	11.58(0.30)	4.56	0.74	5.30	1.18	53%
Oracle	0.12(0.18)	5	0	5	0	100%

**Table 4:** Univariate polynomial logistic regression models of age and log(BMI) fitted separately, with their respective higher order terms.

Variable	Estimate	Std. error	z-value	p-value
Intercept	−3.575	1.168	−3.061	2E–03
Age	0.107	4E–02	2.645	8E–03
Age^2^	−8E-04	4E–04	−2.220	2.6E–02
Intercept	−38.952	8.684	−4.486	7.3E–06
log(BMI)	22.561	5.035	4.480	7.45E–06
log(BMI)^2^	−3.255	0.729	−4.468	7.88E–06

**Table 5:** Estimation results of the categorical clinical variables for the CATHGEN data.

Variable	GPLM-BAR	LASSO	ALASSO
Hypertension	0.368_(0.315)_	0.324_(0.157)_	0.259_(0.093)_
Diabetes	1.180_(0.269)_	1.097_(0.242)_	1.105_(0.261)_
Hypercholesterolomia	1.403_(0.299)_	1.459_(0.275)_	1.399_(0.234)_
Sex	0.361_(0.224)_	0.343_(0.193)_	0.313_(0.219)_
Smoking	0.860_(0.335)_	0.787_(0.301)_	0.797_(0.221)_
HXMI	37.388_(1.786)_	10.620_(0.745)_	10.477_(0.561)_
Race (African)	−0.030_(0.489)_	−0.163_(0.341)_	0.879_(0.584)_
Race (Caucasian)	0.385_(0.458)_	0.544_(0.528)_	0.084_(0.394)_

Male and Asian-American are the reference categories for the variables Sex and Race, respectively. The remaining variables have the non-cases as the reference categories.

## Appendix A. Supplementary data

Supplementary material related to this article can be found online at https://doi.org/10.1016/j.jcmds.2025.100127.
