# Supplementary material for: Broken adaptive ridge method for variable selection in generalized partly linear models with application to the coronary artery disease data
Source: J Comput Math Data Sci. Author manuscript; Available in PMC 2026 Jan 31. (PMC12857884; doi:10.1016/j.jcmds.2025.100127)
Supplement: Supplement [file NIHMS2136907-supplement-Supplement.pdf]

Supplementary Material for "Broken Adaptive Ridge Method for Variable Selection in Generalized Partly Linear Models with Application to the Coronary Artery Disease Data"

Christian Chan, Xiaotian Dai, Thierry Chekouo, Quan Long and Xuewen Lu

Cyclic coordinate descent algorithm

The cyclic coordinate descent algorithm first sets all parameters to some chosen initial value. It solves a one-dimensional optimization problem by estimating the first parameter that minimizes the objective function, while holding the other parameters constant. It then estimates subsequent parameters by solving a one-dimensional problem and holding the other parameters constant. When all parameters are estimated, the iteration is complete and returns to the first parameter for the algorithm to be repeated. Multiple iterations are done over the whole set of parameters until the pre-specified convergence criteria is met. Computing the Hessian matrix or gradient vector, inverting the Hessian matrix, and transposing the gradient vector are not required in the CLG algorithm, as only one-dimensional updates are used. As a result, the CLG algorithm easily scales to high-dimensional data (Wu and Lange, 2008; Simon et al., 2011; Gorst-Rasmussen and Scheike, 2012). It has then been implemented under GLM for massive sample size data (Suchard et al., 2013; Li et al., 2021) and high-dimensional massive sample size Cox PH model (Mittal et al., 2014). Suchard et al. (2013) and Mittal et al. (2014) developed an R package `Cyclops` that incorporates ridge and LASSO regularization using Gaussian and Laplace priors. More specifically, when $s = 0$, the following prior is needed to obtain the initial estimates $\widehat{\boldsymbol{\beta}}^{(0)} : f(\beta_j|\xi_n) \sim N(0, 1/\xi_n)$. For each $s \geq 1$, the reweighted prior $f(\widehat{\beta}_j^{(s)}|\lambda_n) \sim N(0, \widehat{\beta}_j^{(s-1)2}/\lambda_n)$ is used to obtain $\widehat{\boldsymbol{\beta}}^{(s)}$.

The CLG algorithm finds $\{\alpha_j^{(\text{new})}, \beta_j^{(\text{new})}, \gamma_{jk}^{(\text{new})}\}$, where $\{\alpha_j^{(\text{new})}, \beta_j^{(\text{new})}\}$ are the updated estimates of the jth entry of $\boldsymbol{\alpha}$ and $\boldsymbol{\beta}$, and $\gamma_{jk}^{(\text{new})}$ is the updated estimated of the $(j,k)^{th}$ entry of $\boldsymbol{\gamma}$, while keeping the other values of α_j's,β_j's and γ_{jk}'s constant. Suppose we have a tuning parameter $1/\phi_j$. Therefore, when $s \geq 0$, finding $\beta_j^{(\text{new})}$ for $j = 1, \ldots, p$ is equivalent to finding u that minimizes the penalized negative log-likelihood

$$
\begin{aligned}
f(u) = &- \sum_{i=1}^{n} \Bigg\{ y_i \Big(u x_{ij} + \sum_{k=1, j\neq k}^{p} \beta_k x_{ik} + \sum_{k=1}^{q_w} \alpha_k w_{ik} + \sum_{k=1}^{q_z} \sum_{l=0}^{m_k} \gamma_{kl} B_{kl}(z_{ik}, m_k, c_k, u_k) \Big) \\
&- \log \Big(1 + \exp \big\{ u x_{ij} + \sum_{k=1, j\neq k}^{p} \beta_k x_{ik} + \sum_{k=1}^{q_w} \alpha_k w_{ik} + \sum_{k=1}^{q_z} \sum_{l=0}^{m_k} \gamma_{kl} B_{kl}(z_{ik}, m_k, c_k, u_k) \big\} \Big) \Bigg\} \\
&+ \frac{u^2}{2\phi_j}.
\end{aligned} \tag{1}
$$

At the current β_j, one could use the Taylor expansion to approximate $f(u)$ by

$$f(u) \approx f(\beta_j) + f'(\beta_j)(u-\beta_j) + \frac{1}{2}f''(\beta_j)(u-\beta_j)^2.$$

In (1), different penalty terms can used. For example, $\phi_j = 1/\xi_n$ and $\phi_j = (\widehat{\beta}_j^{(s-1)})^2/\lambda_n$ in the GPLM-BAR algorithm. Similarly, finding $\alpha_j^{(\mathrm{new})}$ for $j = 1, \ldots, q_w$ is equivalent to minimizing

$$\begin{aligned} g(u) = &-\sum_{i=1}^{n}\Big\{y_i\Big(\sum_{k=1}^{p}\beta_k x_{ik} + u w_{ij} + \sum_{k=1,j\neq k}^{q_w}\alpha_k w_{ik} + \sum_{k=1}^{q_z}\sum_{l=0}^{m_k}\gamma_{kl}B_{kl}(z_{ik}, m_k, c_k, u_k)\Big) \\ &- \log\Big(1+\exp\Big\{\sum_{k=1}^{p}\beta_k x_{ik} + u w_{ij} + \sum_{k=1,j\neq k}^{q_w}\alpha_k w_{ik} + \sum_{k=1}^{q_z}\sum_{l=0}^{m_k}\gamma_{kl}B_{kl}(z_{ik}, m_k, c_k, u_k)\Big\}\Big)\Big\}, \end{aligned}$$

and finding $\gamma_{jk}^{(\mathrm{new})}$ for $j = 1, \ldots, q_z$ and $k = 1, \ldots, m_j$ is equivalent to minimizing

$$\begin{aligned} h(u) = &-\sum_{i=1}^{n}\Big\{y_i\Big(\sum_{k=1}^{p}\beta_k x_{ik} + \sum_{k=1}^{q_w}\alpha_k w_{ik} + uB_{jk}(z_{ij}, m_j, c_j, u_j) + \sum_{s=1,j\neq s}^{q_z}\sum_{t=0,k\neq t}^{m_s}\gamma_{st}B_{st}(z_{is}, m_s, c_s, u_s)\Big) \\ &- \log\Big(1+\exp\Big\{\sum_{k=1}^{p}\beta_k x_{ik} + \sum_{k=1}^{q_w}\alpha_k w_{ik} + uB_{jk}(z_{ij}, m_j, c_j, u_j) + \sum_{s=1,j\neq s}^{q_z}\sum_{t=0,k\neq t}^{m_s}\gamma_{st}B_{st}(z_{is}, m_s, c_s, u_s)\Big\}\Big)\Big\}. \end{aligned}$$

Suppose the Taylor series is also used to approximate $g(u)$ and $h(u)$, then values of $\{\alpha_j^{(\mathrm{new})}, \beta_j^{(\mathrm{new})}, \gamma_{jk}^{(\mathrm{new})}\}$ can be computed as

$$\beta_j^{(\mathrm{new})} = \beta_j - \frac{f'(\beta_j)}{f''(\beta_j)}, \alpha_j^{(\mathrm{new})} = \alpha_j - \frac{g'(\alpha_j)}{g''(\alpha_j)}, \gamma_{jk}^{(\mathrm{new})} = \gamma_{jk} - \frac{h'(\gamma_{jk})}{h''(\gamma_{jk})}.$$

The first-order and second-order derivations of $f(u)$, $g(u)$ and $h(u)$ are computed as follows. When $s = 0$, $\phi_j = 1/\xi_n$, then the first-order and second-order derivatives are

$$\begin{aligned} f'(u) &= \frac{\partial}{\partial u}f(u)\Big|_{u=\beta_j} \\ &= -\sum_{i=1}^{n}\left\{x_{ij}y_i - \frac{x_{ij}\exp\{ux_{ij} + \sum_{k=1,j\neq k}^{p}\beta_k x_{ik} + \sum_{k=1}^{q_w}\alpha_k w_{ik} + \sum_{k=1}^{q_z}\sum_{l=0}^{m_k}\gamma_{kl}B_{kl}(z_{ik}, m_k, c_k, u_k)\}}{1+\exp\{ux_{ij} + \sum_{k=1,j\neq k}^{p}\beta_k x_{ik} + \sum_{k=1}^{q_w}\alpha_k w_{ik} + \sum_{k=1}^{q_z}\sum_{l=0}^{m_k}\gamma_{kl}B_{kl}(z_{ik}, m_k, c_k, u_k)\}}\right\} \\ &+ 2\xi_n u, \end{aligned}$$

and

$$\begin{aligned} f''(u) &= \frac{\partial^2}{\partial u^2}f(u)\Big|_{u=\beta_j} \\ &= -\sum_{i=1}^{n}\left\{-\frac{x_{ij}^2\exp\{ux_{ij} + \sum_{k=1,j\neq k}^{p}\beta_k x_{ik} + \sum_{k=1}^{q_w}\alpha_k w_{ik} + \sum_{k=1}^{q_z}\sum_{l=0}^{m_k}\gamma_{kl}B_{kl}(z_{ik}, m_k, c_k, u_k)\}}{(1+\exp\{ux_{ij} + \sum_{k=1,j\neq k}^{p}\beta_k x_{ik} + \sum_{k=1}^{q_w}\alpha_k w_{ik} + \sum_{k=1}^{q_z}\sum_{l=0}^{m_k}\gamma_{kl}B_{kl}(z_{ik}, m_k, c_k, u_k)\})^2}\right\} \\ &+ 2\xi_n, \end{aligned}$$

respectively. When $s \geq 1$, $\phi_j = (\widehat{\beta}_j^{(s-1)})^2/\lambda_n$, then

$$\begin{aligned} f'(u) &= \frac{\partial}{\partial u} f(u)\Big|_{u=\beta_j} \\ &= -\sum_{i=1}^{n}\left\{x_{ij}y_i - \frac{x_{ij}\exp\{ux_{ij}+\sum_{k=1,j\neq k}^{p}\beta_k x_{ik}+\sum_{k=1}^{q_w}\alpha_k w_{ik}+\sum_{k=1}^{q_z}\sum_{l=0}^{m_k}\gamma_{kl}B_{kl}(z_{ik},m_k,c_k,u_k)\}}{1+\exp\{ux_{ij}+\sum_{k=1,j\neq k}^{p}\beta_k x_{ik}+\sum_{k=1}^{q_w}\alpha_k w_{ik}+\sum_{k=1}^{q_z}\sum_{l=0}^{m_k}\gamma_{kl}B_{kl}(z_{ik},m_k,c_k,u_k)\}}\right\} \\ &+ \frac{2\lambda_n u}{(\widehat{\beta}_j^{(s-1)})^2}, \end{aligned}$$

and

$$\begin{aligned} f''(u) &= \frac{\partial^2}{\partial u^2} f(u)\Big|_{u=\beta_j} \\ &= -\sum_{i=1}^{n}\left\{ -\frac{x_{ij}^2\exp\{ux_{ij}+\sum_{k=1,j\neq k}^{p}\beta_k x_{ik}+\sum_{k=1}^{q_w}\alpha_k w_{ik}+\sum_{k=1}^{q_z}\sum_{l=0}^{m_k}\gamma_{kl}B_{kl}(z_{ik},m_k,c_k,u_k)\}}{(1+\exp\{ux_{ij}+\sum_{k=1,j\neq k}^{p}\beta_k x_{ik}+\sum_{k=1}^{q_w}\alpha_k w_{ik}+\sum_{k=1}^{q_z}\sum_{l=0}^{m_k}\gamma_{kl}B_{kl}(z_{ik},m_k,c_k,u_k)\})^2}\right\} \\ &+ \frac{2\lambda_n}{(\widehat{\beta}_j^{(s-1)})^2}. \end{aligned}$$

Similarly, the first and second derivatives of $g(u)$ are

$$\begin{aligned} g'(u) &= \frac{\partial}{\partial u} g(u)\Big|_{u=\alpha_j} \\ &= -\sum_{i=1}^{n}\Bigg\{w_{ij}y_i \\ &- \frac{w_{ij}\exp\{\sum_{k=1}^{p}\beta_k x_{ik}++uw_{ij}+\sum_{k=1,j\neq k}^{q_w}\alpha_k w_{ik}+\sum_{k=1}^{q_z}\sum_{l=0}^{m_k}\gamma_{kl}B_{kl}(z_{ik},m_k,c_k,u_k)\}}{1+\exp\{\sum_{k=1}^{p}\beta_k x_{ik}+uw_{ij}+\sum_{k=1,j\neq k}^{q_w}\alpha_k w_{ik}+\sum_{k=1}^{q_z}\sum_{l=0}^{m_k}\gamma_{kl}B_{kl}(z_{ik},m_k,c_k,u_k)\}}\Bigg\}, \end{aligned}$$

and

$$\begin{aligned} g''(u) &= \frac{\partial^2}{\partial u^2} g(u)\Big|_{u=\alpha_j} \\ &= -\sum_{i=1}^{n}\left\{ -\frac{w_{ij}^2\exp\{\sum_{k=1}^{p}\beta_k x_{ik}+uw_{ij}+\sum_{k=1,j\neq k}^{q_w}\alpha_k w_{ik}+\sum_{k=1}^{q_z}\sum_{l=0}^{m_k}\gamma_{kl}B_{kl}(z_{ik},m_k,c_k,u_k)\}}{(1+\exp\{\sum_{k=1}^{p}\beta_k x_{ik}+uw_{ij}+\sum_{k=1,j\neq k}^{q_w}\alpha_k w_{ik}+\sum_{k=1}^{q_z}\sum_{l=0}^{m_k}\gamma_{kl}B_{kl}(z_{ik},m_k,c_k,u_k)\})^2}\right\}. \end{aligned}$$

Finally, the first and second derivatives of $h(u)$ are

$$\begin{aligned} h'(u) &= \frac{\partial}{\partial u} h(u)\Big|_{u=\gamma_{jk}} \\ &= -\sum_{i=1}^{n}\left\{\ddot{B}_{jk}y_i - \frac{\ddot{B}_{jk}\exp\{\sum_{k=1}^{p}\beta_k x_{ik}+\sum_{k=1}^{q_w}\alpha_k w_{ik}+u\ddot{B}_{jk}+\sum_{s=1,j\neq s}^{q_z}\sum_{t=0,k\neq t}^{m_s}\gamma_{st}\ddot{B}_{st}\}}{1+\exp\{\sum_{k=1}^{p}\beta_k x_{ik}+\sum_{k=1}^{q_w}\alpha_k w_{ik}+u\ddot{B}_{jk}+\sum_{s=1,j\neq s}^{q_z}\sum_{t=0,k\neq t}^{m_s}\gamma_{st}\ddot{B}_{st}\}}\right\}, \end{aligned}$$

and

$$h''(u) = \frac{\partial^2}{\partial u^2} h(u)\Big|_{u=\gamma_{jk}}$$
$$= - \sum_{i=1}^{n} \Big\{ - \frac{\ddot{B}_{jk}^2 \exp\{\sum_{k=1}^{p} \beta_k x_{ik} + \sum_{k=1}^{q_w} \alpha_k w_{ik} + u\ddot{B}_{jk} + \sum_{s=1,j\neq s}^{q_z} \sum_{t=0,k\neq t}^{m_s} \gamma_{st}\ddot{B}_{st}\}}{(1 + \exp\{\sum_{k=1}^{p} \beta_k x_{ik} + \sum_{k=1}^{q_w} \alpha_k w_{ik} + u\ddot{B}_{jk} + \sum_{s=1,j\neq s}^{q_z} \sum_{t=0,k\neq t}^{m_s} \gamma_{st}\ddot{B}_{st}\})^2} \Big\},$$

where $\ddot{B}_{jk} = B_{jk}(z_{ik}, m_k, c_k, u_k)$ and $\ddot{B}_{st} = B_{st}(z_{is}, m_s, c_s, u_s)$.

Additional results of the simulation studies

More simulation results for the logistic partly linear model

In this section, we present more simulation results that could not be included in the main article, by presenting the following tables, where Scenarios 1 and 2 are for the logistic partly linear model and Scenario 5 is for the Poisson partly linear model.

Table 1: Estimation results of non-zero elements in $\boldsymbol{\beta}$ in Scenario 1. Standard deviations are in parentheses.

Method	Bias($\widehat{\beta}_1$)	Bias($\widehat{\beta}_2$)	Bias($\widehat{\beta}_{p-2}$)	Bias($\widehat{\beta}_{p-1}$)	Bias($\widehat{\beta}_p$)
			$n = 600, p = 300$		
BAR(AIC)	-0.01(0.15)	0.01(0.14)	0.01(0.13)	-0.03(0.14)	-0.01(0.14)
BAR(BIC)	-0.23(0.15)	0.23(0.15)	0.26(0.13)	-0.29(0.24)	-0.17(0.22)
LASSO	-0.54(0.11)	0.54(0.11)	0.53(0.10)	-0.44(0.10)	-0.35(0.11)
ALASSO	-0.32(0.16)	0.32(0.16)	0.33(0.15)	-0.30(0.16)	-0.20(0.15)
SCAD	-0.92(0.06)	0.92(0.05)	0.93(0.05)	-0.71(0.04)	-0.68(0.05)
MCP	-0.86(0.02)	0.86(0.02)	0.86(0.02)	-0.65(0.02)	-0.64(0.02)
Oracle	0.06(0.15)	-0.06(0.14)	-0.07(0.13)	0.04(0.13)	0.05(0.14)
			$n = 800, p = 300$		
BAR(AIC)	-0.01(0.13)	0.01(0.13)	0.02(0.12)	-0.02(0.11)	-0.01(0.12)
BAR(BIC)	-0.16(0.11)	0.17(0.12)	0.18(0.11)	-0.17(0.15)	-0.14(0.14)
LASSO	-0.48(0.10)	0.49(0.10)	0.49(0.09)	-0.40(0.09)	-0.33(0.09)
ALASSO	-0.28(0.15)	0.29(0.15)	0.29(0.14)	-0.26(0.14)	-0.20(0.13)
SCAD	-0.91(0.05)	0.91(0.05)	0.92(0.05)	-0.71(0.04)	-0.68(0.04)
MCP	-0.86(0.02)	0.86(0.02)	0.86(0.02)	-0.65(0.02)	-0.65(0.02)
Oracle	0.04(0.12)	-0.04(0.12)	-0.04(0.11)	0.03(0.12)	0.02(0.11)
			$n = 600, p = 450$		
BAR(AIC)	0.01(0.15)	-0.02(0.16)	0.01(0.14)	-0.01(0.14)	0.01(0.13)
BAR(BIC)	-0.21(0.15)	0.21(0.17)	0.27(0.17)	-0.27(0.25)	-0.18(0.20)
LASSO	-0.53(0.11)	0.53(0.12)	0.55(0.10)	-0.44(0.10)	-0.36(0.09)
ALASSO	-0.26(0.15)	0.26(0.16)	0.29(0.14)	-0.25(0.15)	-0.17(0.13)
SCAD	-0.91(0.06)	0.91(0.06)	0.93(0.05)	-0.71(0.05)	-0.69(0.04)
MCP	-0.86(0.02)	0.86(0.02)	0.86(0.02)	-0.65(0.03)	-0.64(0.02)
Oracle	0.08(0.15)	-0.08(0.16)	-0.05(0.14)	0.05(0.13)	0.05(0.13)
			$n = 800, p = 450$		
BAR(AIC)	-0.01(0.12)	0.01(0.12)	0.01(0.11)	-0.02(0.12)	-0.01(0.12)
BAR(BIC)	-0.17(0.11)	0.17(0.11)	0.19(0.11)	-0.18(0.16)	-0.14(0.13)
LASSO	-0.50(0.09)	0.50(0.09)	0.50(0.08)	-0.41(0.09)	-0.33(0.09)
ALASSO	-0.26(0.15)	0.26(0.15)	0.27(0.14)	-0.24(0.15)	-0.17(0.13)
SCAD	-0.91(0.05)	0.91(0.05)	0.92(0.05)	-0.70(0.05)	-0.68(0.04)
MCP	-0.86(0.02)	0.86(0.02)	0.86(0.02)	-0.64(0.02)	-0.64(0.02)
Oracle	0.04(0.12)	-0.04(0.13)	-0.04(0.13)	0.03(0.12)	0.03(0.11)

Table 2: Estimation results of non-zero elements in $\boldsymbol{\beta}$ for Scenario 2. Standard deviations are in parentheses.

Method	Bias($\widehat{\beta}_1$)	Bias($\widehat{\beta}_2$)	Bias($\widehat{\beta}_{p-2}$)	Bias($\widehat{\beta}_{p-1}$)	Bias($\widehat{\beta}_p$)
			$n = 600, p = 300$		
BAR(AIC)	-0.02(0.16)	0.08(0.15)	0.03(0.15)	-0.12(0.18)	0.02(0.18)
BAR(BIC)	-0.46(0.12)	0.46(0.11)	0.46(0.12)	-0.35(0.09)	-0.30(0.15)
LASSO	-0.52(0.11)	0.41(0.10)	0.52(0.10)	-0.33(0.09)	-0.35(0.10)
ALASSO	-0.27(0.15)	0.33(0.14)	0.26(0.14)	-0.27(0.13)	-0.15(0.13)
SCAD	-0.92(0.05)	0.50(0.01)	0.92(0.05)	-0.40(0.01)	-0.69(0.04)
MCP	-0.85(0.02)	0.44(0.04)	0.85(0.02)	-0.37(0.04)	-0.63(0.02)
Oracle	0.06(0.11)	-0.02(0.10)	-0.06(0.11)	0.03(0.11)	0.05(0.12)
			$n = 800, p = 300$		
BAR(AIC)	-0.03(0.11)	0.05(0.11)	0.03(0.11)	-0.07(0.15)	-0.01(0.13)
BAR(BIC)	-0.24(0.16)	0.36(0.17)	0.24(0.16)	-0.36(0.13)	-0.07(0.18)
LASSO	-0.48(0.09)	0.38(0.09)	0.47(0.08)	-0.30(0.09)	-0.32(0.08)
ALASSO	-0.25(0.12)	0.30(0.12)	0.24(0.12)	-0.25(0.12)	-0.13(0.11)
SCAD	-0.92(0.05)	0.50(0.01)	0.91(0.04)	-0.40(0.01)	-0.69(0.04)
MCP	-0.85(0.02)	0.43(0.03)	0.85(0.02)	-0.36(0.03)	-0.63(0.02)
Oracle	0.03(0.09)	-0.01(0.10)	-0.04(0.09)	0.01(0.10)	0.03(0.10)
			$n = 600, p = 450$		
BAR(AIC)	-0.03(0.15)	0.07(0.14)	0.03(0.15)	-0.11(0.19)	0.01(0.17)
BAR(BIC)	-0.30(0.13)	0.44(0.13)	0.29(0.09)	-0.37(0.08)	-0.13(0.15)
LASSO	-0.54(0.10)	0.42(0.10)	0.54(0.09)	-0.34(0.08)	-0.37(0.10)
ALASSO	-0.26(0.15)	0.32(0.14)	0.26(0.13)	-0.26(0.14)	-0.15(0.14)
SCAD	-0.92(0.05)	0.50(0.01)	0.92(0.05)	-0.40(0.01)	-0.69(0.04)
MCP	-0.85(0.02)	0.44(0.04)	0.86(0.02)	-0.37(0.04)	-0.63(0.02)
Oracle	0.03(0.12)	-0.03(0.11)	-0.05(0.11)	0.02(0.12)	0.02(0.11)
			$n = 800, p = 450$		
BAR(AIC)	-0.02(0.11)	0.04(0.11)	0.01(0.12)	-0.05(0.14)	0.01(0.15)
BAR(BIC)	-0.38(0.18)	0.38(0.18)	0.39(0.17)	-0.31(0.14)	-0.24(0.18)
LASSO	-0.49(0.09)	0.38(0.09)	0.48(0.09)	-0.31(0.09)	-0.32(0.09)
ALASSO	-0.22(0.12)	0.27(0.12)	0.21(0.12)	-0.23(0.13)	-0.11(0.13)
SCAD	-0.92(0.05)	0.50(0.01)	0.91(0.05)	-0.40(0.01)	-0.69(0.04)
MCP	-0.85(0.02)	0.43(0.03)	0.85(0.02)	-0.36(0.03)	-0.63(0.02)
Oracle	0.02(0.10)	-0.02(0.10)	-0.04(0.10)	0.02(0.10)	0.03(0.10)

Table 3: Estimation results of $\boldsymbol{\alpha}$ for Scenario 1. Standard deviations are in parentheses.

Method	Bias($\widehat{\alpha}_1$)	Bias($\widehat{\alpha}_2$)	Bias($\widehat{\alpha}_3$)	Bias($\widehat{\alpha}_4$)	Bias($\widehat{\alpha}_5$)
$n = 600, p = 300$					
BAR(AIC)	0.02(0.26)	-0.01(0.23)	-0.01(0.22)	0.01(0.22)	-0.03(0.24)
BAR(BIC)	-0.10(0.23)	0.05(0.20)	0.05(0.19)	-0.08(0.20)	0.10(0.21)
LASSO	-0.23(0.20)	0.12(0.18)	0.12(0.17)	-0.18(0.18)	0.23(0.19)
ALASSO	-0.14(0.22)	0.07(0.20)	0.07(0.19)	-0.11(0.20)	0.14(0.22)
SCAD	-0.76(0.03)	0.52(0.04)	0.52(0.03)	-0.55(0.03)	0.95(0.04)
MCP	-0.76(0.03)	0.52(0.03)	0.52(0.03)	-0.55(0.03)	0.95(0.03)
Oracle	0.06(0.26)	-0.03(0.24)	-0.03(0.22)	0.04(0.23)	-0.06(0.24)
$n = 800, p = 300$					
BAR(AIC)	0.02(0.20)	-0.01(0.19)	-0.03(0.19)	0.01(0.19)	-0.02(0.21)
BAR(BIC)	-0.08(0.18)	0.04(0.17)	0.03(0.17)	-0.07(0.17)	0.08(0.19)
LASSO	-0.22(0.16)	0.11(0.15)	0.10(0.15)	-0.17(0.15)	0.22(0.17)
ALASSO	-0.13(0.18)	0.07(0.17)	0.05(0.17)	-0.10(0.17)	0.13(0.20)
SCAD	-0.76(0.03)	0.52(0.03)	0.52(0.03)	-0.55(0.03)	0.95(0.03)
MCP	-0.76(0.03)	0.52(0.03)	0.52(0.03)	0.55(0.03)	0.95(0.03)
Oracle	0.04(0.20)	-0.02(0.20)	-0.04(0.19)	0.02(0.20)	-0.04(0.21)
$n = 600, p = 450$					
BAR(AIC)	0.06(0.23)	-0.02(0.22)	-0.03(0.25)	0.05(0.24)	-0.04(0.24)
BAR(BIC)	-0.08(0.20)	0.06(0.19)	0.04(0.21)	-0.06(0.21)	0.11(0.21)
LASSO	-0.22(0.18)	0.11(0.17)	0.11(0.18)	-0.16(0.19)	0.24(0.19)
ALASSO	-0.10(0.21)	0.06(0.19)	0.05(0.21)	-0.06(0.22)	0.12(0.22)
SCAD	-0.76(0.03)	0.52(0.04)	0.52(0.04)	-0.55(0.04)	0.95(0.04)
MCP	-0.76(0.03)	0.52(0.03)	0.52(0.04)	-0.55(0.03)	0.95(0.04)
Oracle	0.09(0.23)	-0.02(0.22)	-0.02(0.25)	0.05(0.24)	0.10(0.25)
$n = 800, p = 450$					
BAR(AIC)	0.02(0.21)	-0.01(0.21)	-0.01(0.18)	0.01(0.18)	-0.02(0.20)
BAR(BIC)	-0.08(0.19)	0.04(0.19)	0.05(0.16)	-0.07(0.17)	0.08(0.18)
LASSO	-0.23(0.17)	0.11(0.17)	0.12(0.15)	-0.18(0.15)	0.23(0.16)
ALASSO	-0.12(0.20)	0.06(0.19)	0.06(0.17)	-0.09(0.17)	0.12(0.19)
SCAD	-0.76(0.03)	0.52(0.03)	0.52(0.03)	-0.55(0.03)	0.95(0.03)
MCP	-0.76(0.03)	0.52(0.03)	0.52(0.03)	-0.55(0.03)	0.95(0.03)
Oracle	0.04(0.21)	-0.02(0.21)	-0.01(0.18)	0.03(0.19)	-0.04(0.20)

Table 4: Estimation results of $\boldsymbol{\alpha}$ for Scenario 2. Standard deviations are in parentheses.

Method	Bias($\widehat{\alpha}_1$)	Bias($\widehat{\alpha}_2$)	Bias($\widehat{\alpha}_3$)	Bias($\widehat{\alpha}_4$)	Bias($\widehat{\alpha}_5$)
			$n = 600, p = 300$		
BAR(AIC)	0.01(0.21)	0.01(0.22)	-0.02(0.20)	0.02(0.22)	-0.02(0.20)
BAR(BIC)	-0.11(0.19)	0.06(0.20)	0.04(0.19)	-0.07(0.21)	0.09(0.18)
LASSO	-0.21(0.19)	0.10(0.20)	0.09(0.19)	-0.15(0.20)	0.19(0.18)
ALASSO	-0.11(0.19)	0.06(0.21)	0.04(0.19)	-0.07(0.21)	0.09(0.18)
SCAD	-0.76(0.04)	0.52(0.04)	0.52(0.04)	-0.54(0.04)	0.94(0.03)
MCP	-0.75(0.04)	0.52(0.03)	0.52(0.03)	-0.54(0.04)	0.94(0.03)
Oracle	0.04(0.21)	-0.01(0.22)	-0.03(0.21)	0.05(0.22)	-0.06(0.20)
			$n = 800, p = 300$		
BAR(AIC)	0.01(0.18)	0.01(0.17)	0.01(0.18)	0.02(0.17)	-0.03(0.17)
BAR(BIC)	-0.10(0.17)	0.06(0.17)	0.05(0.17)	-0.06(0.16)	0.07(0.16)
LASSO	-0.19(0.17)	0.10(0.16)	0.10(0.17)	-0.14(0.15)	0.17(0.15)
ALASSO	-0.10(0.17)	0.06(0.16)	0.06(0.17)	-0.07(0.15)	0.08(0.15)
SCAD	-0.75(0.03)	0.52(0.03)	0.52(0.03)	-0.54(0.03)	0.94(0.03)
MCP	-0.75(0.03)	0.52(0.03)	0.52(0.03)	-0.54(0.03)	0.94(0.03)
Oracle	0.03(0.18)	-0.01(0.17)	-0.01(0.18)	0.03(0.17)	-0.05(0.17)
			$n = 600, p = 450$		
BAR(AIC)	0.02(0.21)	-0.01(0.21)	0.01(0.21)	0.01(0.19)	-0.04(0.21)
BAR(BIC)	-0.11(0.20)	0.05(0.20)	0.06(0.20)	-0.07(0.17)	0.08(0.20)
LASSO	-0.21(0.19)	0.10(0.20)	0.12(0.19)	-0.15(0.16)	0.18(0.19)
ALASSO	-0.10(0.20)	0.04(0.20)	0.07(0.19)	-0.07(0.18)	0.08(0.20)
SCAD	-0.76(0.03)	0.52(0.03)	0.52(0.03)	-0.54(0.04)	0.94(0.04)
MCP	-0.76(0.03)	0.52(0.03)	0.52(0.03)	-0.54(0.04)	0.94(0.03)
Oracle	0.05(0.21)	-0.03(0.21)	-0.02(0.21)	0.05(0.19)	0.08(0.21)
			$n = 800, p = 450$		
BAR(AIC)	0.02(0.21)	-0.01(0.21)	-0.01(0.18)	0.03(0.18)	-0.01(0.20)
BAR(BIC)	-0.08(0.19)	0.04(0.19)	0.05(0.16)	-0.05(0.17)	0.08(0.18)
LASSO	-0.19(0.16)	0.11(0.16)	0.09(0.14)	-0.14(0.14)	0.20(0.16)
ALASSO	-0.08(0.20)	0.04(0.18)	0.04(0.16)	-0.05(0.16)	0.09(0.18)
SCAD	-0.75(0.03)	0.52(0.03)	0.52(0.03)	-0.54(0.03)	0.94(0.03)
MCP	-0.75(0.03)	0.52(0.03)	0.52(0.03)	-0.54(0.03)	0.94(0.03)
Oracle	0.03(0.19)	-0.01(0.21)	0.01(0.20)	0.05(0.18)	0.04(0.18)

Table 5: Mean and median computation time (measured in seconds) for one Monte-Carlo replication in Scenario 1. SD: standard deviation.

Method	Mean (SD)	Median
$n = 600, p = 300$		
BAR	1.61(0.34)	1.56
LASSO	2.65(0.29)	2.63
ALASSO	3.99(0.35)	3.96
SCAD	13.50(6.48)	12.23
MCP	23.828(3.37)	24.375
$n = 800, p = 300$		
BAR	2.12(0.23)	2.14
LASSO	7.20(2.17)	6.78
ALASSO	7.84(1.04)	7.75
SCAD	19.43(11.7)	16.29
MCP	13.97(11.30)	9.965
$n = 600, p = 450$		
BAR	2.22(0.41)	2.17
LASSO	2.39(0.14)	2.38
ALASSO	4.35(0.18)	4.35
SCAD	13.97(6.32)	12.63
MCP	13.97(6.33)	12.63
$n = 800, p = 450$		
BAR	2.74(0.42)	2.71
LASSO	4.26(0.32)	4.23
ALASSO	6.61(0.40)	6.59
SCAD	18.35(10.80)	14.95
MCP	13.65(10.21)	10.31

Table 6: Variable selection and estimation results over 500 replications for Scenario 3, where $\beta_0 = \{1, -1, 0, \ldots, 0, -1, 0.75, 0.75\}^\top$. Standard deviations of the MMSE are in parentheses. MMSE: Median Mean Squared Error; TP: True Positive; FP: False Positive; MS: Model Size; MC: Total Misclalssification = FN + FP; TM: Frequency of True Model selected. Bold values indicate the best method.

Method	MMSE	TP	FP	MS	MC	TM
$n = 600, p = 2000$						
BAR(AIC)	0.35(0.19)	**5**	2.99	7.99	2.99	5%
BAR(BIC)	**0.31(0.28)**	4.74	**0**	**4.74**	**0.26**	**75%**
BAR(HBIC)	3.44(0.36)	0.34	**0**	0.34	4.66	0%
LASSO	1.32(0.26)	**5**	6.84	11.84	6.84	10%
ALASSO	0.56(0.28)	**5**	36.35	41.35	36.35	3%
SCAD	3.03(0.16)	4.73	4.77	9.50	5.04	51%
MCP	2.54(0.08)	4.94	2.27	7.21	2.33	28%
Oracle	0.08(0.08)	5	0	5	0	100%
$n = 800, p = 2000$						
BAR(AIC)	0.35(0.19)	**5**	4.79	9.79	4.79	4%
BAR(BIC)	**0.28(0.23)**	4.96	**0**	**4.96**	**0.21**	**96%**
BAR(HBIC)	3.44(0.52)	2.26	**0**	2.26	2.74	0%
LASSO	1.30(0.27)	**5**	5.73	10.73	4.2	18%
ALASSO	0.47(0.23)	**5**	35.38	40.38	35.38	5%
SCAD	3.09(0.15)	4.87	2.40	7.27	2.53	52%
MCP	2.70(0.05)	4.99	1.29	6.28	1.30	21%
Oracle	0.07(0.08)	5	0	5	0	100%
$n = 600, p = 4000$						
BAR(AIC)	**0.19(0.14)**	4.99	1.09	6.08	1.10	31%
BAR(BIC)	0.28(0.28)	4.76	**0**	**4.76**	**0.24**	**77%**
BAR(HBIC)	3.44(0.23)	0.15	**0**	0.15	4.85	0%
LASSO	1.41(0.26)	4.99	7.78	12.77	7.79	9%
ALASSO	0.68(0.32)	**5**	60.23	65.23	60.23	0%
SCAD	3.03(0.16)	4.80	1.01	5.81	1.21	48%
MCP	2.54(0.07)	4.94	2.45	7.39	2.51	21%
Oracle	0.08(0.07)	5	0	5	0	100%
$n = 800, p = 4000$						
BAR(AIC)	**0.22(0.16)**	4.99	1.46	6.45	1.47	21%
BAR(BIC)	0.26(0.25)	4.77	**0**	**4.77**	**0.23**	**77%**
BAR(HBIC)	3.44(0.34)	0.26	**0**	3.70	1.82	0%
LASSO	1.38(0.28)	4.98	5.22	10.20	5.24	18%
ALASSO	0.58(0.30)	**5**	51.25	56.25	51.25	2%
SCAD	3.08(0.16)	4.84	4.30	9.14	4.46	38%
MCP	2.70(0.05)	4.96	4.03	8.99	4.07	10%
Oracle	0.07(0.10)	5	0	5	0	100%

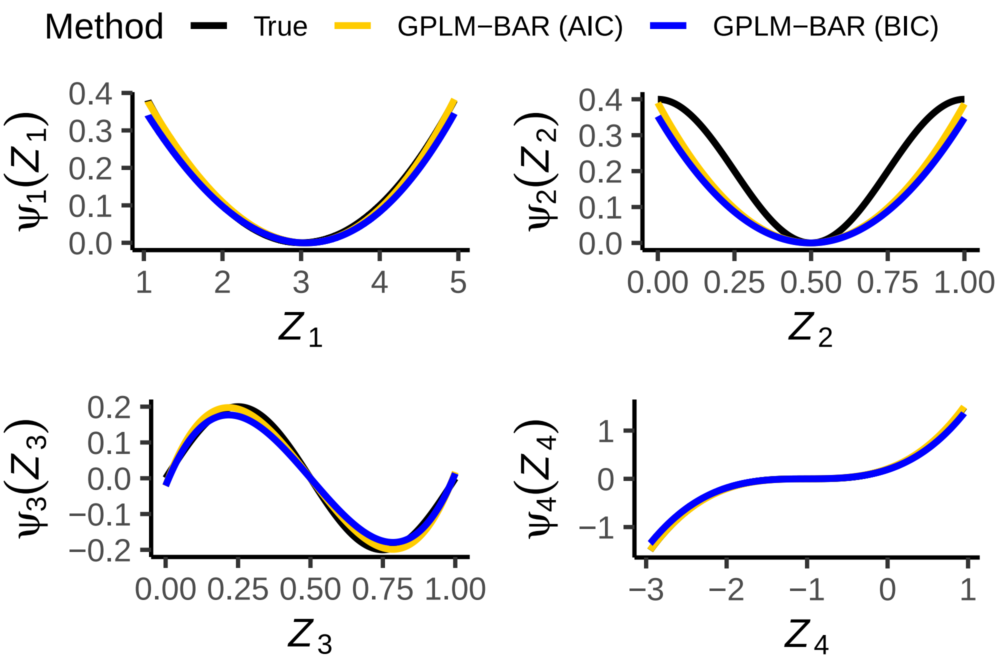

Figure 1: Estimated nonlinear covariate effects $\psi_j, j = 1, 2, 3, 4$ for $p = 300$ and $n = 800$ for Scenario 1. The true functions are: $\psi_1(z_1) = 0.1(z_1 - 3)^2$ (top left), $\psi_2(z_2) = 0.2(\cos(2\pi z_2) + 1)$ (top right), $\psi_3(z_3) = 0.2\sin(2\pi z_3)$ (bottom left), $\psi_4 z_4 = 0.2(z_4 + 1)^3$ (bottom right).

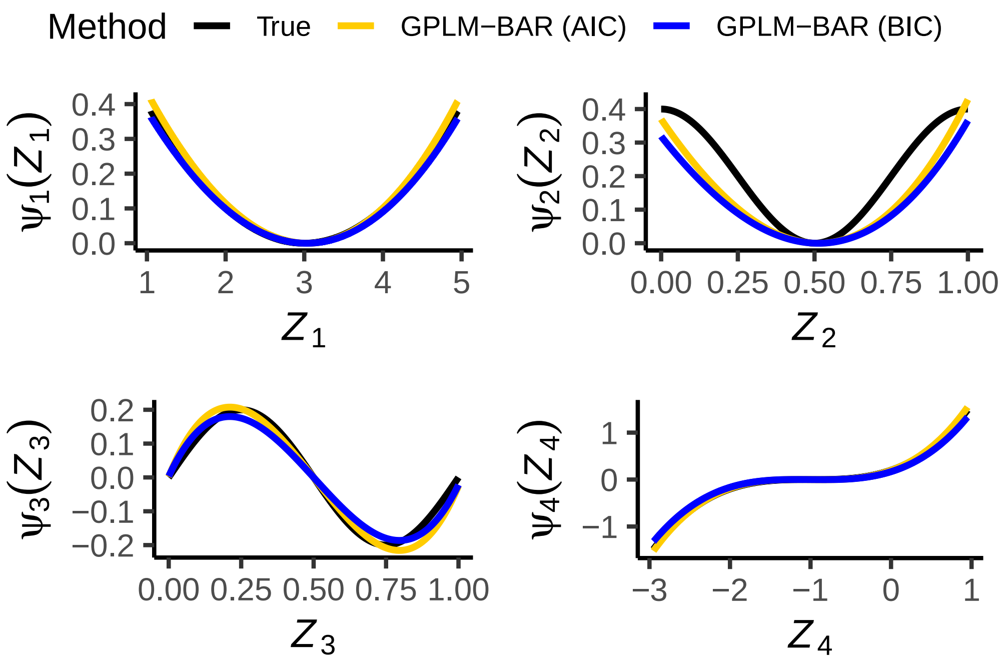

Figure 2: Estimated nonlinear covariate effects $\psi_j, j = 1, 2, 3, 4$ for $p = 450$ and $n = 600$ for Scenario 1. The true functions are: $\psi_1(z_1) = 0.1(z_1 - 3)^2$ (top left), $\psi_2(z_2) = 0.2(\cos(2\pi z_2) + 1)$ (top right), $\psi_3(z_3) = 0.2 \sin(2\pi z_3)$ (bottom left), $\psi_4 z_4 = 0.2(z_4 + 1)^3$ (bottom right).

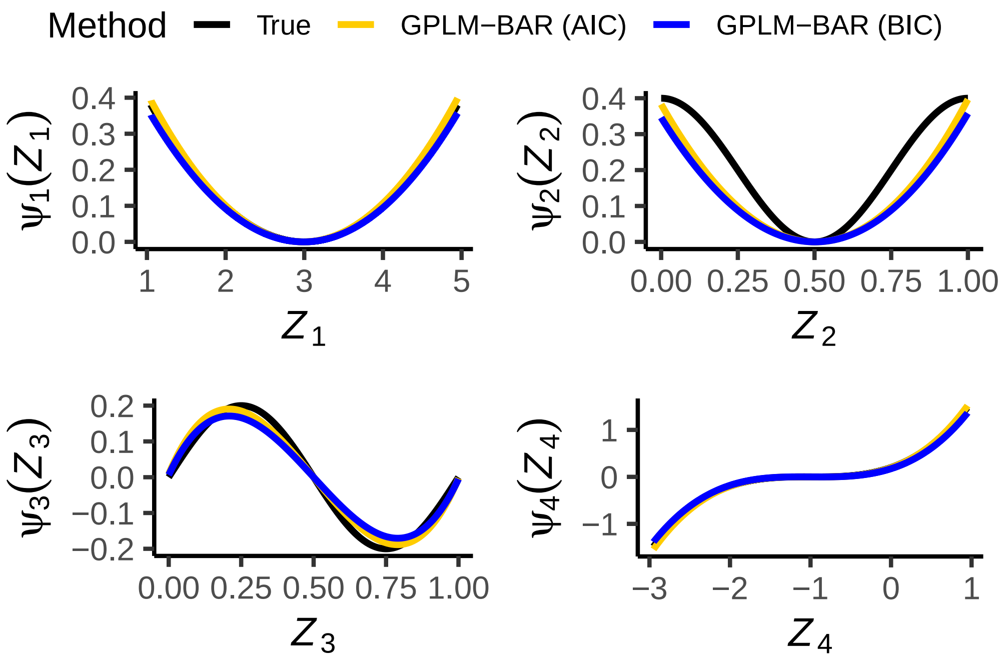

Figure 3: Estimated nonlinear covariate effects $\psi_j, j = 1, 2, 3, 4$ for $p = 450$ and $n = 800$ for Scenario 1. The true functions are: $\psi_1(z_1) = 0.1(z_1 - 3)^2$ (top left), $\psi_2(z_2) = 0.2(\cos(2\pi z_2) + 1)$ (top right), $\psi_3(z_3) = 0.2 \sin(2\pi z_3)$ (bottom left), $\psi_4 z_4 = 0.2(z_4 + 1)^3$ (bottom right).

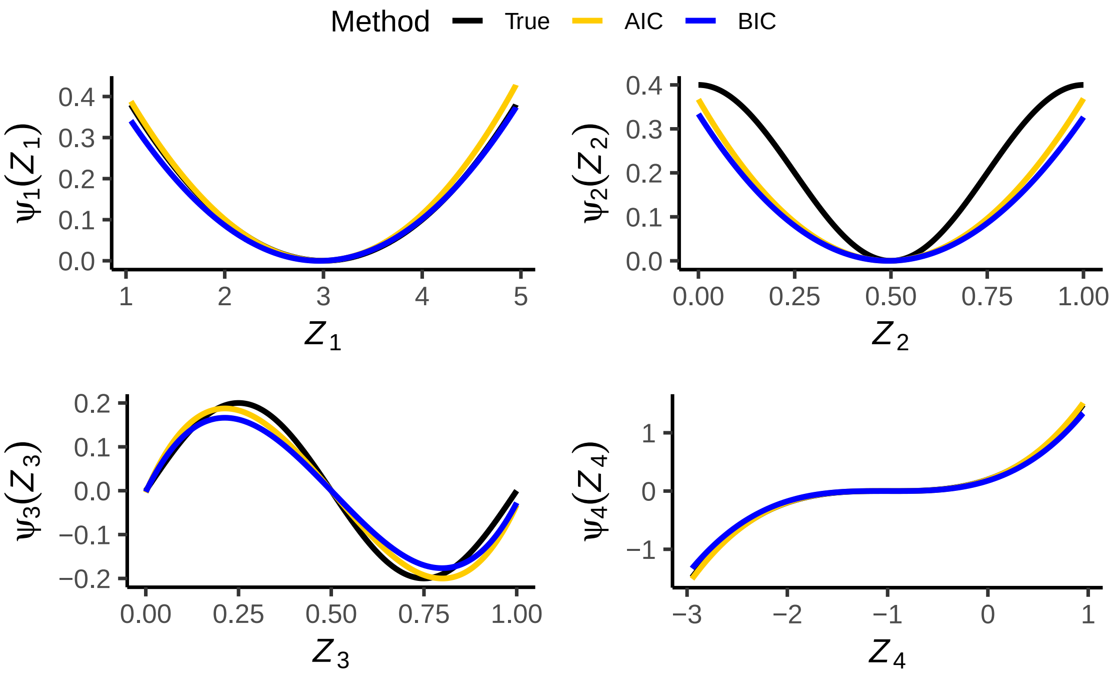

Figure 4: Estimated nonlinear covariate effects $\psi_j, j = 1, 2, 3, 4$ for $p = 300$ and $n = 600$ for Scenario 2. The true functions are: $\psi_1(z_1) = 0.1(z_1-3)^2$ (top left), $\psi_2(z_2) = 0.2(\cos(2\pi z_2)+1)$ (top right), $\psi_3(z_3) = 0.2\sin(2\pi z_3)$ (bottom left), $\psi_4 z_4 = 0.2(z_4+1)^3$ (bottom right).

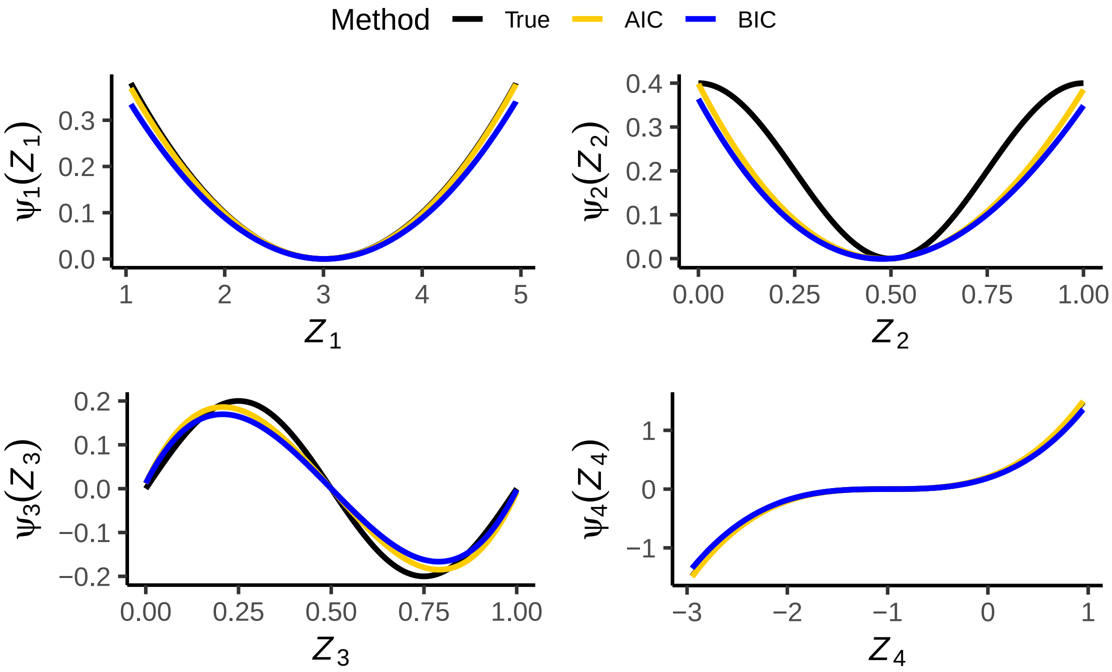

Figure 5: Estimated nonlinear covariate effects $\psi_j, j = 1, 2, 3, 4$ for $p = 300$ and $n = 800$ for Scenario 2. The true functions are: $\psi_1(z_1) = 0.1(z_1-3)^2$ (top left), $\psi_2(z_2) = 0.2(\cos(2\pi z_2)+1)$ (top right), $\psi_3(z_3) = 0.2\sin(2\pi z_3)$ (bottom left), $\psi_4 z_4 = 0.2(z_4+1)^3$ (bottom right).

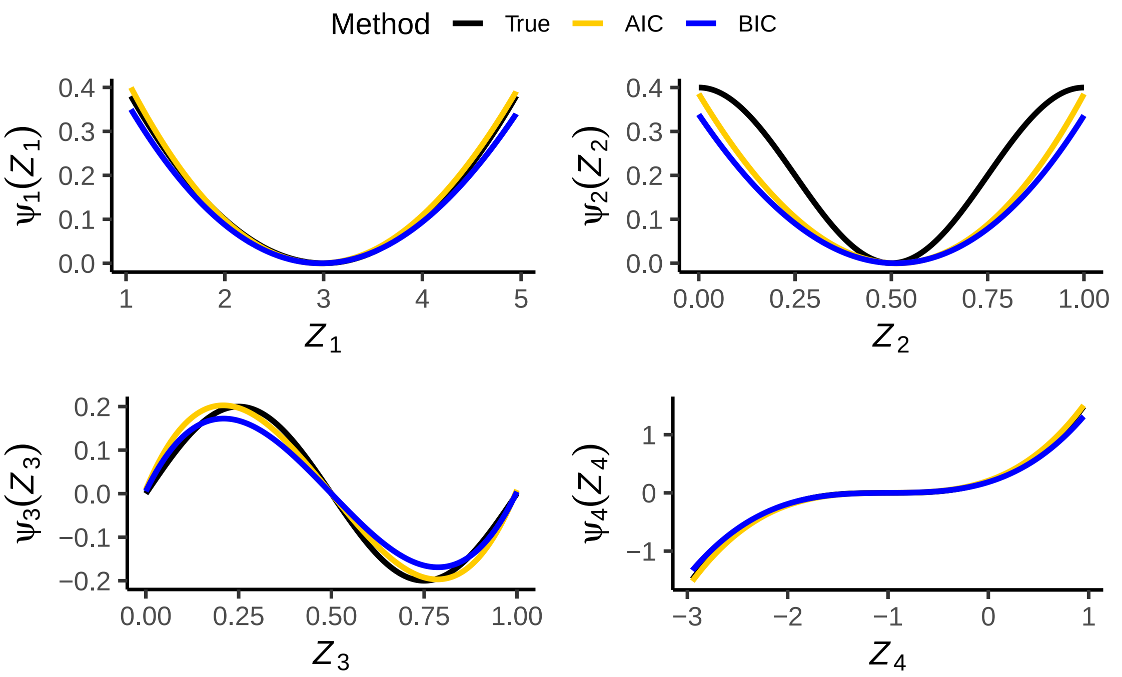

Figure 6: Estimated nonlinear covariate effects $\psi_j, j = 1, 2, 3, 4$ for $p = 450$ and $n = 600$ for Scenario 2. The true functions are: $\psi_1(z_1) = 0.1(z_1-3)^2$ (top left), $\psi_2(z_2) = 0.2(\cos(2\pi z_2)+1)$ (top right), $\psi_3(z_3) = 0.2\sin(2\pi z_3)$ (bottom left), $\psi_4 z_4 = 0.2(z_4 + 1)^3$ (bottom right).

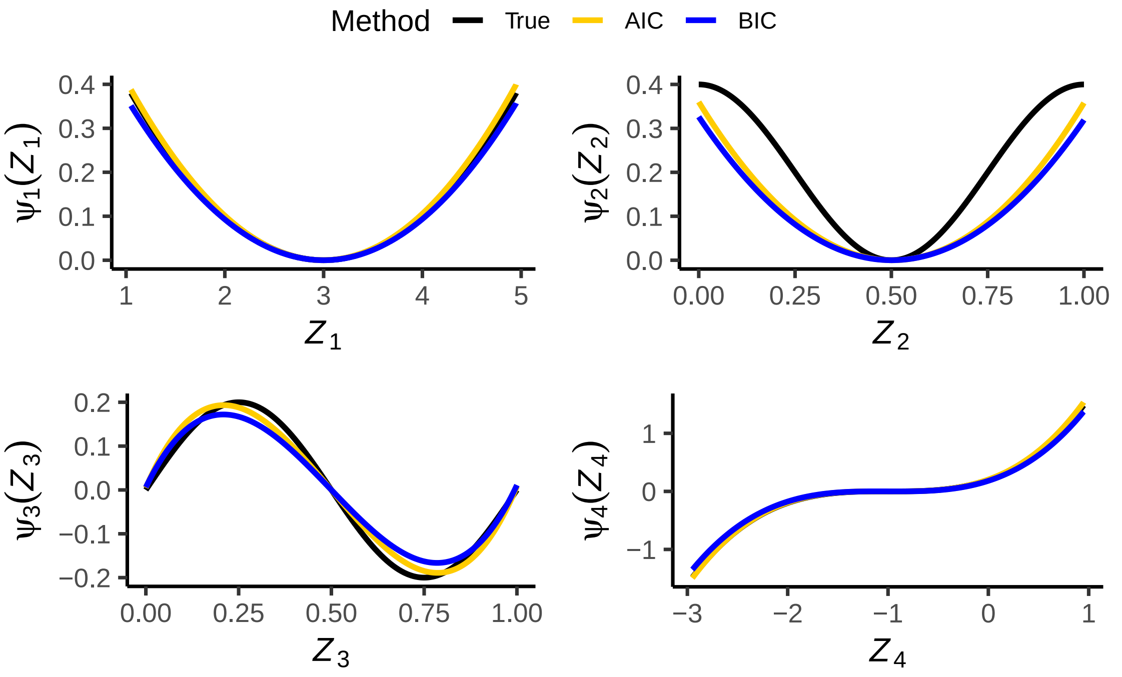

Figure 7: Estimated nonlinear covariate effects $\psi_j, j = 1, 2, 3, 4$ for $p = 450$ and $n = 800$ for Scenario 2. The true functions are: $\psi_1(z_1) = 0.1(z_1-3)^2$ (top left), $\psi_2(z_2) = 0.2(\cos(2\pi z_2)+1)$ (top right), $\psi_3(z_3) = 0.2\sin(2\pi z_3)$ (bottom left), $\psi_4 z_4 = 0.2(z_4 + 1)^3$ (bottom right).

Scenario 4: CATHGEN-based simulations

We perform a simulation study based on the CATHGEN data, where the motivation is to examine the performance of our proposed method, in comparison with existing methods, under the correlation structure of our data. Before conducting the simulation study, we first have to screen the data to reduce dimension. The final screened set is 1313 observations and 1242 SNPs. Then, we use the GPLM-BAR to determine which SNPs are relevant to the binary response, where 19 SNPs are selected. In the simulation set-up, we set the effect size of the identified SNPs as four times the true estimated effect size. We do this because the original effects are too small for the model to distinguish them. We do not change the effect size of the categorical and continuous variables. We also do not change the coefficients of the Bernstein polynomials.

Table 7: Variable selection and estimation results over 200 replications for Scenario 4. Standard deviation of the MMSE are in parentheses. MMSE: Median Mean Squared Error; TP: True Positive; FP: False Positive; MS: Model Size; MC: Total Misclalssification = FN + FP; TM: Frequency of True Model selected. Bold values indicate the best method.

Method	MMSE	TP	FP	MS	MC	TM
BAR(AIC)	0.57(0.25)	18.84	2.22	21.06	2.38	9%
BAR(BIC)	6.75(0.98)	12.41	0.07	12.48	6.66	0%
LASSO	5.24(0.65)	18.97	52.73	71.70	52.76	0%
ALASSO	2.82(0.63)	18.89	27.48	45.58	27.59	0%
Oracle	0.30(0.26)	19	0	19	0	100%

From Table 7, the GPLM-BAR method with AIC penalty has the lowest average number of FP and MC. Using the GPLM-BAR with BIC penalty, the average number of TP decreases due to the large size of the tuning parameter λ_n. The Lasso and Adaptive Lasso methods produce very large false positives. In Table 8, the GPLM-BAR with AIC penalty produces the least biased estimates of the relevant SNPs. In Figure 8, we observe that the estimation of the non-linear effects of age and log(BMI) is also better when the AIC penalty, as compared to the BIC penalty, is used.

Table 8: Averaged estimates of the relevant SNPs identified in the real data analysis, performed over 200 replication for Scenario 4, along with the clinical and demographical variables. Standard deviations are in parentheses.

	True	BAR-AIC	BAR-BIC	LASSO	ALASSO	Oracle
rs1407961	-1.024	-0.922(0.136)	-0.444(0.134)	-0.432(0.075)	-0.630(0.099)	-1.088(0.144)
rs8037353	1.017	0.898(0.121)	0.277(0.192)	0.376(0.072)	0.567(0.098)	1.099(0.157)
rs7107322	1.015	0.909(0.129)	0.300(0.176)	0.387(0.078)	0.560(0.105)	1.097(0.139)
rs2387952	-1.336	-1.228(0.129)	-0.709(0.077)	-0.621(0.081)	-0.843(0.102)	-1.410(0.153)
rs6680365	-0.918	-0.830(0.118)	-0.370(0.131)	-0.401(0.069)	-0.555(0.096)	0.980(0.131)
rs3136558	-0.906	-0.806(0.116)	-0.363(0.154)	-0.372(0.067)	-0.530(0.091)	0.961(0.135)
rs4131888	0.512	0.419(0.137)	0.008(0.049)	0.180(0.070)	0.253(0.106)	0.543(0.118)
rs3845439	1.106	0.936(0.276)	0.411(0.154)	0.366(0.123)	0.533(0.168)	1.182(0.136)
rs769449	0.773	0.701(0.104)	0.200(0.174)	0.338(0.063)	0.479(0.083)	0.833(0.125)
rs9549675	-1.092	-0.968(0.127)	-0.461(0.109)	-0.438(0.075)	-0.636(0.100)	-1.169(0.157)
rs2805543	-1.203	-1.091(0.123)	-0.538(0.093)	-0.531(0.085)	-0.731(0.106)	-1.275(0.144)
rs821292	-0.781	-0.666(0.136)	-0.033(0.096)	-0.247(0.076)	-0.362(0.117)	-0.840(0.119)
rs12612481	0.770	0.698(0.116)	0.266(0.161)	0.329(0.075)	0.448(0.104)	0.824(0.108)
rs7188981	0.905	0.823(0.120)	0.402(0.136)	0.398(0.074)	0.560(0.101)	0.975(0.128)
rs9932172	0.997	0.903(0.120)	0.483(0.083)	0.435(0.075)	0.591(0.104)	1.044(0.138)
rs9282537	0.763	0.658(0.129)	0.045(0.117)	0.256(0.074)	0.358(0.119)	0.826(0.123)
rs244072	-0.608	-0.509(0.113)	0	-0.162(0.066)	-0.234(0.103)	-0.660(0.136)
rs11859718	0.760	0.675(0.138)	0.279(0.176)	0.316(0.071)	0.440(0.010)	0.811(0.136)
rs17585580	-0.603	-0.491(0.159)	0	-0.153(0.070)	-0.197(0.118)	-0.648(0.136)
Hypertension	0.368	0.324(0.211)	0.228(0.131)	0.224(0.133)	0.246(0.161)	0.409(0.254)
Diabetes	1.180	1.136(0.216)	0.769(0.137)	0.712(0.137)	0.855(0.161)	1.260(0.299)
Hypercholesterolomia	1.403	1.294(0.218)	0.867(0.128)	0.879(0.132)	0.992(0.158)	1.483(0.258)
Sex	0.361	0.325(0.205)	0.149(0.123)	0.153(0.125)	0.205(0.151)	0.392(0.229)
Smoking	0.860	0.798(0.240)	0.431(0.145)	0.431(0.143)	0.546(0.138)	0.938(0.235)
HXMI	37.388	42.379(1.097)	38.919(1.716)	11.130(0.310)	12.368(0.435)	26.677(0.803)
Race(African)	-0.030	0.031(0.436)	0.308(0.283)	0.114(0.263)	0.116(0.322)	-0.050(0.474)
Race(Caucasian)	0.385	0.357(0.378)	0.016(0.225)	0.123(0.223)	0.236(0.274)	0.412(0.411)

Scenario 5: Poisson partly linear model

In this scenario, we present the details of a simulation study for the Poisson partly linear model. Let $q_w = 5$ and $q_z = 4$. The true values of $\boldsymbol{\beta}$ is $\boldsymbol{\beta}_0 = \{1, -0.75, 0, \dots, 0, -1, 0.75, -0.75\}^\top$, and the true values of $\boldsymbol{\alpha}$ is $\boldsymbol{\alpha}_0 = \{0.75, -0.5, -0.5, 0.75, -1\}^\top$. We generate $\mathbf{X}, \mathbf{W}$, and $\mathbf{Z}$ from the same distributions used in Scenarios 1 and 2. By setting $\psi_1(z_{i1}) = 0.1(z_{i1} - 3)^2$, $\psi_2(z_{i2}) = 0.2(\cos(2\pi z_{i2}) + 1)$, $\psi_3(z_{i3}) = 0.2\sin(2\pi z_{i3})$, and $\psi_4(z_{i4}) = 0.1(z_{i4} + 1)^3$, we generate y_i from a Poisson distribution with a rate $\lambda_i = \exp\{\boldsymbol{\beta}^\top \mathbf{x}_i + \boldsymbol{\alpha}^\top \mathbf{w}_i + \sum_{j=1}^4 \psi_j(z_{ij})\}$. From Table 9, the selection accuracy of GPLM-BAR method is better than the LASSO and Adaptive LASSO methods. We set the number of basis $m_j = 3$, for $j = 1, 2, 3, 4$. We also report the values of the individual estimates of $\boldsymbol{\alpha}$ and $\boldsymbol{\beta}$ in Tables 11 and 10, respectively, where the individual BAR estimates are also the least biased among all methods.

Table 9: Variable selection results over 200 replications for Scenario 5. Standard deviations are in parentheses. MMSE: Median Mean Squared Error; TP: True Positive; FP: False Positive; MS: Model Size; MC: Total Misclalssification = FN + FP; TM: Frequency of True Model selected. Bold values indicate the best method.

Method	MMSE	TP	FP	MS	MC	TM
$n = 600, p = 300$						
BAR(AIC)	0.003(0.003)	5	0.90	5.90	0.90	46%
BAR(BIC)	0.002(0.001)	5	0	5	0	100%
LASSO	0.368(0.967)	4	0.67	4.67	1.67	37%
ALASSO	0.106(0.418)	4.87	0	4.87	0.13	96%
Oracle	0.002(0.001)	5	0	5	0	100%
$n = 800, p = 300$						
BAR(AIC)	0.003(0.002)	5	1.09	6.09	1.09	38%
BAR(BIC)	0.001(0.001)	5	0.01	5.01	0.01	99.5%
LASSO	0.365(0.572)	4.7	0.34	5.04	0.64	66%
ALASSO	0.407(0.320)	4.96	0	4.96	0.04	98%
Oracle	0.001(0.001)	5	0	5	0	100%
$n = 600, p = 450$						
BAR(AIC)	0.004(0.004)	5	0.87	5.87	0.87	44%
BAR(BIC)	0.002(0.002)	5	0	5	0	100%
LASSO	0.321(1.064)	3.75	0.90	4.65	2.15	28%
ALASSO	0.100 (0.525)	4.80	0	4.80	0.20	96%
Oracle	0.002(0.002)	5	0	5	0	100%
$n = 800, p = 450$						
BAR(AIC)	0.003(0.003)	5	1.18	6.18	1.16	34%
BAR(BIC)	0.001(0.001)	5	0	5	0	100%
LASSO	0.310(1.101)	3.65	0.38	4.03	1.73	47%
ALASSO	0.102(0.305)	4.95	0	4.95	0.05	98%
Oracle	0.001(0.001)	5	0	5	0	100%

Table 10: Estimation results of non-zero entries in $\boldsymbol{\beta}$ for Scenario 5. Standard deviations are in parentheses.

Method	Bias($\widehat{\beta}_1$)	Bias($\widehat{\beta}_2$)	Bias($\widehat{\beta}_{p-2}$)	Bias($\widehat{\beta}_{p-1}$)	Bias($\widehat{\beta}_p$)
			$n = 600, p = 300$		
BAR(AIC)	-0.001(0.021)	0.002(0.021)	0.003(0.024)	-0.001(0.023)	0.002(0.022)
BAR(BIC)	-0.005(0.020)	0.006(0.021)	0.007(0.023)	-0.006(0.022)	0.006(0.022)
LASSO	-0.440(0.286)	0.385(0.193)	0.438(0.285)	-0.425(0.177)	0.374(0.200)
ALASSO	-0.179(0.141)	0.204(0.130)	0.189(0.148)	-0.257(0.158)	0.206(0.138)
Oracle	0(0.021)	0(0.020)	0.002(0.023)	0.001(0.022)	0(0.022)
			$n = 800, p = 300$		
BAR(AIC)	0.0006(0.018)	-0.002(0.019)	0.002(0.02)	-0.001(0.018)	0.002(0.017)
BAR(BIC)	-0.002(0.018)	0.002(0.019)	0.005(0.02)	-0.004(0.017)	0.005(0.017)
LASSO	-0.355(0.173)	0.327(0.125)	0.349(0.171)	-0.378(0.118)	0.331(0.124)
ALASSO	-0.244(0.127)	0.278(0.148)	0.245(0.125)	-0.349(0.179)	0.291(0.151)
Oracle	0.001(0.018)	-0.002(0.019)	0.001(0.020)	0.001(0.017)	0.001(0.017)
			$n = 600, p = 450$		
BAR(AIC)	0.003(0.022)	0.001(0.020)	0.003(0.025)	0.001(0.025)	0.003(0.021)
BAR(BIC)	-0.001(0.022)	0.004(0.020)	0.006(0.024)	-0.004(0.024)	0.008(0.021)
LASSO	-0.459(0.318)	0.399(0.213)	0.454(0.320)	-0.43(0.197)	0.396(0.213)
ALASSO	-0.177(0.175)	0.195(0.128)	0.179(0.175)	-0.238(0.136)	0.198(0.132)
Oracle	0.004(0.022)	-0.001(0.020)	0.001(0.024)	0.003(0.024)	0.002(0.021)
			$n = 800, p = 450$		
BAR(AIC)	0.001(0.020)	-0.001(0.017)	-0.001(0.019)	-0.001(0.019)	-0.001(0.019)
BAR(BIC)	-0.001(0.020)	0.002(0.017)	0.001(0.018)	-0.004(0.019)	0.002(0.019)
LASSO	-0.469(0.327)	0.393(0.224)	0.462(0.330)	-0.430(0.203)	0.391(0.224)
ALASSO	-0.186(0.117)	0.215(0.134)	0.188(0.117)	-0.284(0.166)	0.220(0.133)
Oracle	0.002(0.020)	-0.002(0.017)	-0.002(0.018)	0.001(0.019)	-0.002(0.019)

Table 11: Estimation results of $\boldsymbol{\alpha}$ for Scenario 5. Standard deviations are in parentheses.

Method	Bias($\widehat{\alpha}_1$)	Bias($\widehat{\alpha}_2$)	Bias($\widehat{\alpha}_3$)	Bias($\widehat{\alpha}_4$)	Bias($\widehat{\alpha}_5$)
			$n = 600, p = 300$		
BAR(AIC)	-0.001(0.021)	0.002(0.021)	0.003(0.024)	-0.001(0.023)	0.002(0.022)
BAR(BIC)	-0.005(0.020)	0.006(0.021)	0.007(0.023)	-0.006(0.022)	0.006(0.022)
LASSO	-0.440(0.286)	0.385(0.193)	0.438(0.285)	-0.425(0.177)	0.374(0.200)
ALASSO	-0.179(0.141)	0.204(0.130)	0.189(0.148)	-0.257(0.158)	0.206(0.138)
Oracle	0(0.021)	0(0.020)	0.002(0.023)	0.001(0.022)	0(0.022)
			$n = 800, p = 300$		
BAR(AIC)	0.0004(0.037)	0.002(0.037)	-0.001(0.035)	0.004(0.034)	-0.001(0.034)
BAR(BIC)	0.0003(0.037)	0.002(0.036)	0(0.035)	0.004(0.033)	-0.0002(0.035)
LASSO	-0.085(0.204)	0.067(0.157)	0.056(0.164)	-0.080(0.204)	0.107(0.253)
ALASSO	-0.032(0.134)	0.042(0.135)	0.016(0.125)	-0.033(0.136)	0.035(0.133)
Oracle	0.001(0.037)	0.002(0.036)	-0.0003(0.035)	0.005(0.032)	-0.001(0.035)
			$n = 600, p = 450$		
BAR(AIC)	0.003(0.044)	-0.001(0.040)	0.005(0.043)	-0.004(0.045)	0.002(0.044)
BAR(BIC)	0.0003(0.044)	0.001(0.039)	0.006(0.042)	-0.003(0.045)	0.003(0.043)
LASSO	-0.242(0.314)	0.152(0.220)	0.146(0.228)	-0.228(0.319)	0.291(0.424)
ALASSO	-0.066(0.161)	0.040(0.119)	0.037(0.123)	-0.065(0.161)	0.071(0.208)
Oracle	0.002(0.044)	0.0004(0.040)	0.005(0.042)	-0.002(0.045)	0.002(0.043)
			$n = 800, p = 450$		
BAR(AIC)	0.002(0.035)	-0.005(0.034)	0.0003(0.035)	0.001(0.036)	-0.006(0.038)
BAR(BIC)	0.002(0.034)	-0.005(0.034)	0.001(0.034)	0.001(0.036)	-0.005(0.038)
LASSO	-0.239(0.322)	0.152(0.229)	0.159(0.223)	-0.228(0.331)	0.304(0.433)
ALASSO	-0.021(0.119)	0.018(0.104)	0.018(0.115)	-0.011(0.123)	0.023(0.124)
Oracle	0.003(0.034)	-0.005(0.034)	0.001(0.034)	0.002(0.036)	-0.006(0.038)

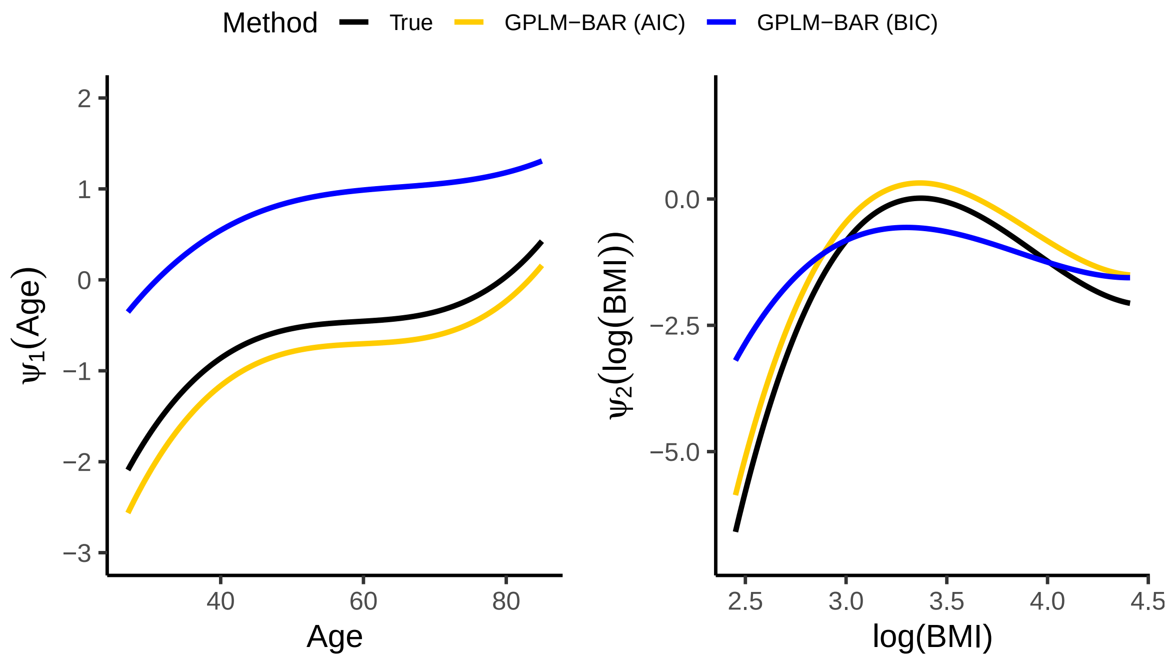

Figure 8: Estimation of the non-linear effects of age and log(BMI) based on the simulated CATHGEN data.

More real data analysis results

Table 12: SNPs and its associated gene selected by the GPLM-BAR method.

SNP	Gene
rs1407961	ZMYM2
rs8037353	GABRG3
rs7107322	PRCP
rs2387952	ADARB2
rs6680365	CAMTA1
rs3136558	IL1B
rs4131888	SLC7A11
rs3845439	CACNA1E
rs769449	APOE
rs9549675	F10
rs2805543	ADARB2
rs821292	GFOD1
rs12612481	PDE11A
rs7188981	RBFOX1
rs9932172	RBFOX1
rs9282537	ABCA1
rs244072	ADA
rs11859718	CDH13
rs17585580	CNTN5

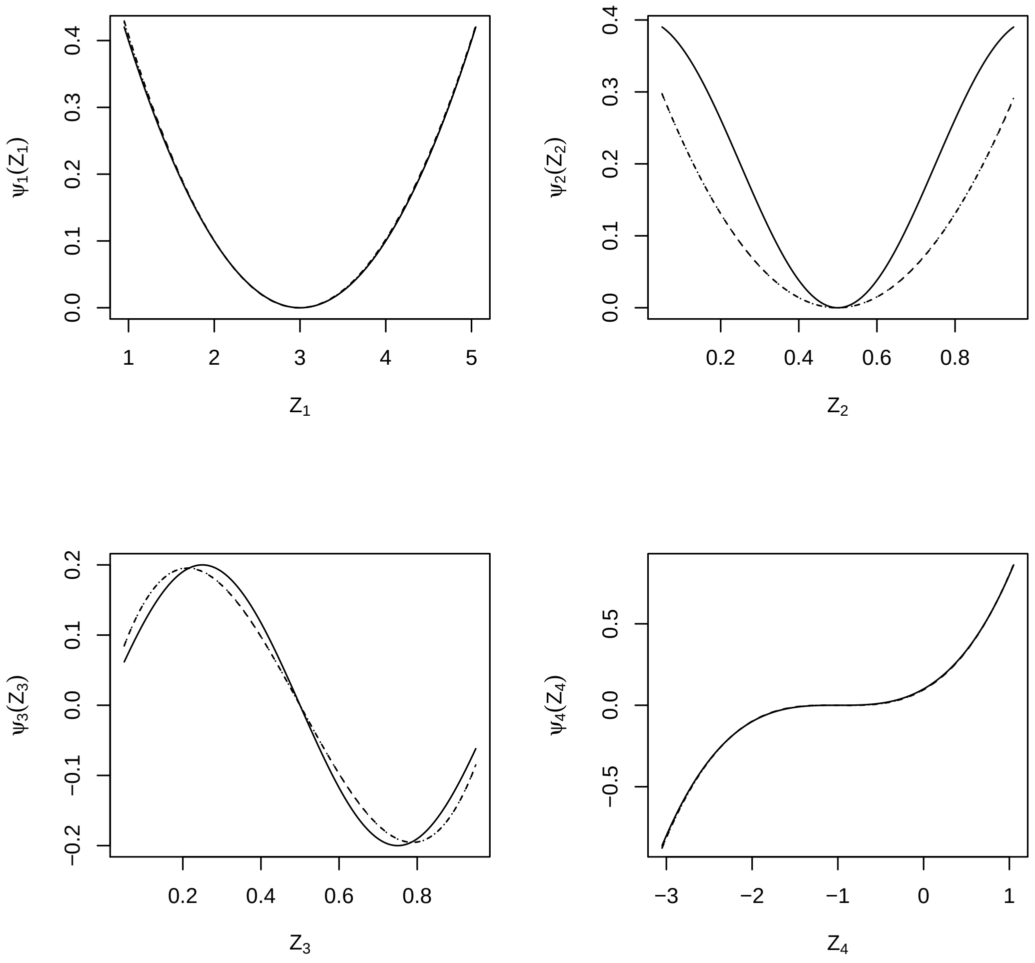

Figure 9: Estimated nonlinear covariate effects $\psi_j, j = 1, 2, 3, 4$ for $p = 300$ and $n = 600$ for Scenario 5. The dashed line represents the GPLM-BAR with AIC penalty, and the dotted line represents GPLM-BAR method with BIC penalty.

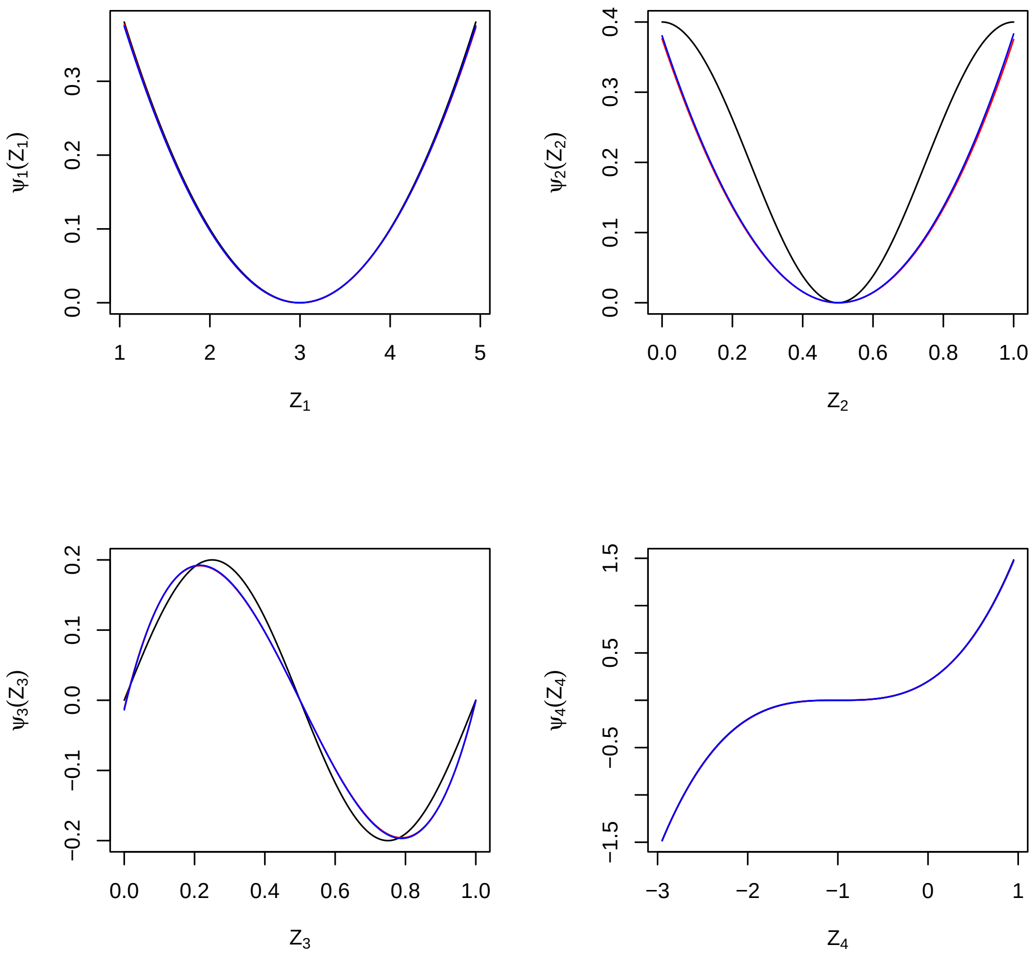

Figure 10: Estimated nonlinear covariate effects $\psi_j, j = 1, 2, 3, 4$ for $p = 450$ and $n = 600$ for Scenario 5. The blue line represents the GPLM-BAR with AIC penalty, and the red line represents GPLM-BAR method with BIC penalty.

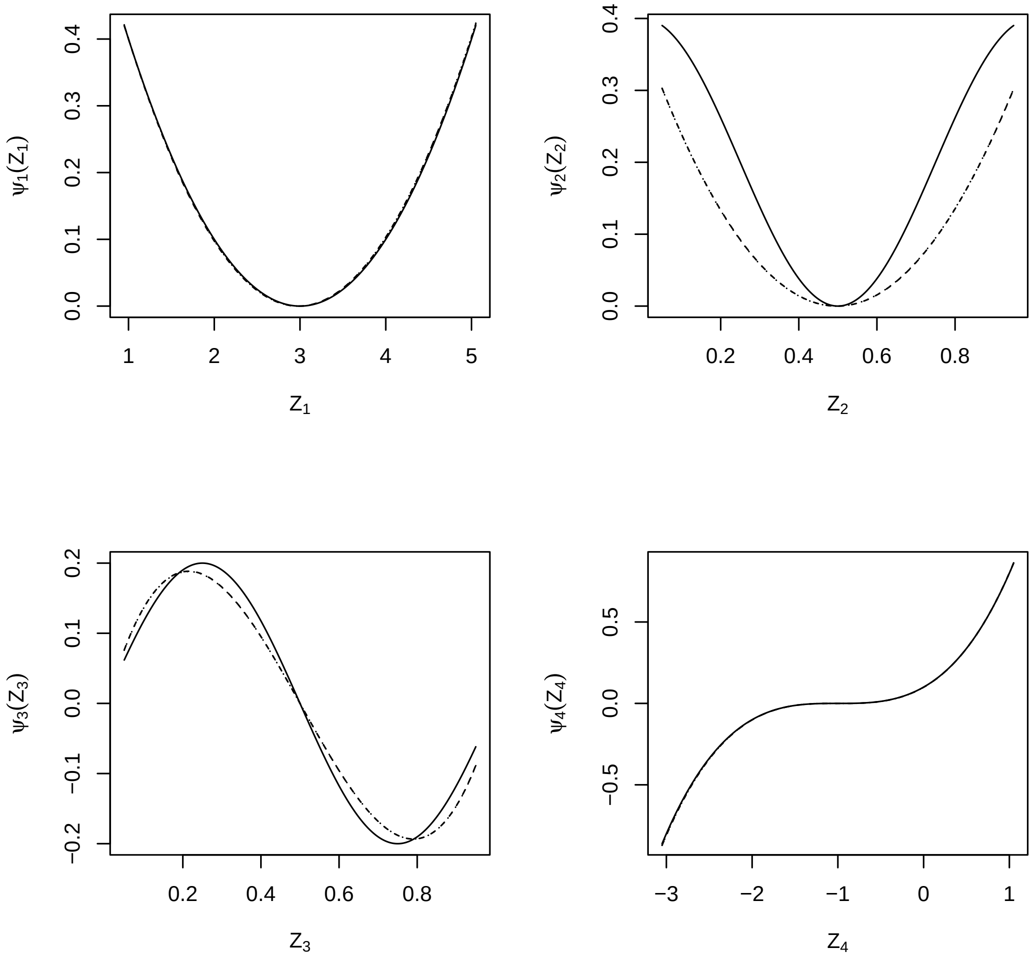

Figure 11: Estimated nonlinear covariate effects $\psi_j, j = 1, 2, 3, 4$ for $p = 300$ and $n = 800$ for Scenario 5. The dashed line represents the GPLM-BAR with AIC penalty, and the dotted line represents GPLM-BAR method with BIC penalty.

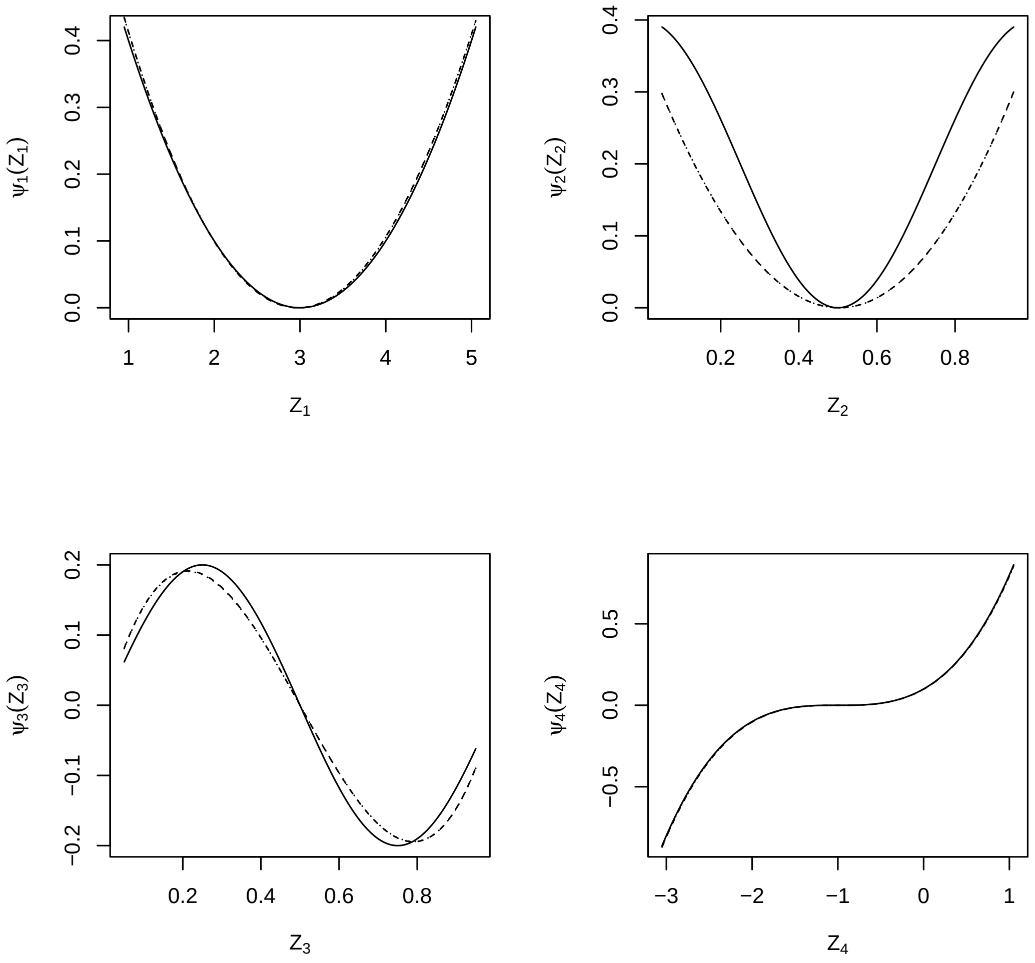

Figure 12: Estimated nonlinear covariate effects $\psi_j, j = 1, 2, 3, 4$ for $p = 450$ and $n = 800$ for Scenario 5. The dashed line represents the GPLM-BAR with AIC penalty, and the dotted line represents GPLM-BAR method with BIC penalty.

References

Gorst-Rasmussen, A. and Scheike, T. H. (2012). Coordinate descent methods for the penalized semiparametric additive hazards model. *Journal of Statistical Software*, **47**, 1–17.

Li, N., Peng, X., Kawaguchi, E., Suchard, M.A., and Li, G. (2021). A scalable surrogate L_0 sparse regression method for generalized linear models with applications to large scale data. *Journal of Statistical Planning and Inference*, **213**, 262–281.

Mittal, S., Madigan, D., Burd R. S., and Suchard M. A. (2014). High-dimensional, massive sample-size Cox proportional hazards regression for survival analysis. *Biostatistics*, **15**, 207–221.

Simon, N., Friedman, J., Hastie, T., and Tibshirani, R. (2011). Regularization paths for Cox's proportional hazards model via coordinate descent. *Journal of Statistical Software*, **39**, 1–13.

Suchard, M.A., Simpson, S.E., Zorych, I., Ryan, P, and Madigan, D. (2013). Massive parallelization of serial inference algorithms for a complex generalized linear model. *ACM Transactions on Modeling and Computer Simulation (TOMACS)*, **23**, 1–17.

Wu, T. and Lange, K. (2008). Coordinate descent algorithms for Lasso penalized regression. *The Annals of Applied Statistics*, **2**, 224–244.
